# Targeted Radionuclide Therapy Activates Prodrugs for Treating Metastasis

**DOI:** 10.1021/acscentsci.4c01369

**Published:** 2024-12-05

**Authors:** Zhibin Guo, Xuanyu Wang, Yi Han, Siyong Shen, Peng Tian, Yuchen Hu, Zexuan Ding, Qunfeng Fu, Zhibo Liu

**Affiliations:** † Beijing National Laboratory for Molecular Sciences, Radiochemistry and Radiation Chemistry Key Laboratory of Fundamental Science, College of Chemistry and Molecular Engineering, Peking University, Beijing 100871, China; ‡ China Institute of Atomic Energy, Institute of Nuclear Technology, Beijing 102413, China; § Changping Laboratory, Beijing 102206, China; ∥ Peking University-Tsinghua University Centre for Life Sciences, Peking University, Beijing 100871, China; ⊥ Key Laboratory of Carcinogenesis and Translational Research (Ministry of Education/Beijing), NMPA Key Laboratory for Research and Evaluation of Radiopharmaceuticals (National Medical Products Administration), Department of Nuclear Medicine, Peking University Cancer Hospital & Institute, Beijing 100142, China

## Abstract

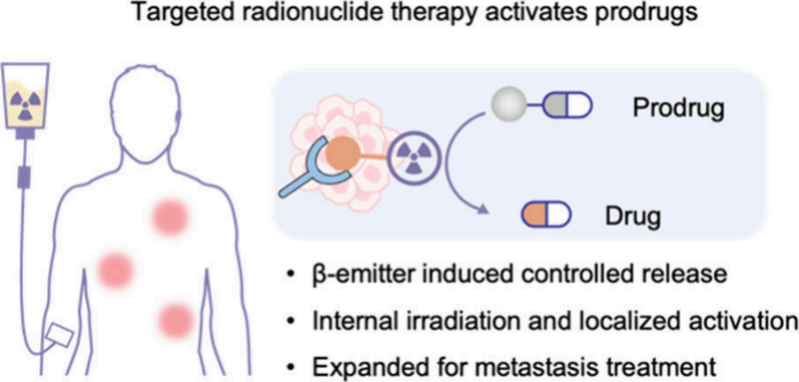

Over 90% of cancer patients succumb to metastasis, yet conventional frontline therapy struggles to halt the progression of metastatic tumors. Targeted radionuclide therapy, which delivers radiation precisely to tumor sites, shows promise for treating metastasis. The rational design of a prodrug activation platform using radionuclides would be an ideal approach to synergize chemotherapy with targeted radionuclide therapy, yet it has not been established. Here, we present targeted radionuclide therapy-induced cleavage chemistry that enables the controlled release of oxaliplatin and its axis ligands from oxaliplatin(IV) complexes in living systems. Of note, this strategy demonstrates feasibility over clinically relevant β-emitting radionuclides and exhibits dose dependence. These advantages were taken into account, and a Lutetium-177-activatable platinum(IV) based prodrug system was designed that could achieve localized activation at the tumor site with high efficiency, thereby suppressing subcutaneous and metastatic 4T1 tumors. In summary, our approach highlights the potential of radionuclides as reaction switches, bridging the gap between the radiotherapy-induced reaction and internal radiation. It may provide a new perspective for future combination therapy.

## Abstract 2

A targeted radionuclide therapy-induced cleavage strategy is designed for *in vivo* release of oxaliplatin and axial ligands from Pt(IV) compounds, offering a potential approach for treating metastasis.

## Introduction

**Scheme 1:**
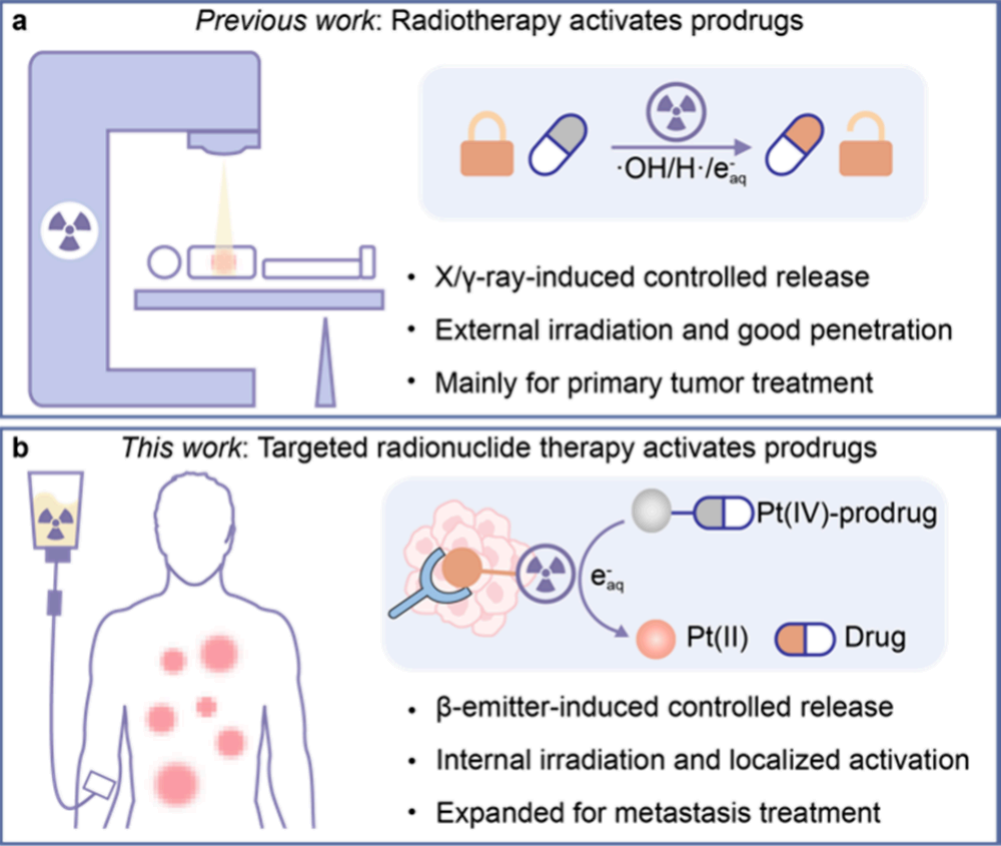
Targeted Radionuclide Therapy-Induced Prodrug Activation in Metastatic Tumor (a) Radiotherapy-induced prodrug activation for treating primary tumor. Reactive particles such as hydroxyl radical (·OH), hydrogen radical (H·)m and hydrated electron (e^–^_aq_) generated by radiolysis could induce the cleavage of the specific chemical bond to release the corresponding antitumor agent. (b) In this work, we designed a targeted radionuclide therapy activatable Pt(IV) prodrug platform, which utilizes e^–^_aq_ generated by internal radiation emitted by radionuclides to release Pt(II) complexes and active drugs at the tumor site, effectively treating metastasis.

For decades, cancer has been a leading cause of death worldwide.^1^ The combination of radiotherapy and chemotherapy has benefited over 60% of cancer patients, but severe side effects and dose limitations restrict its wider clinical use.^2,3^ Recent research highlights the potential of radiotherapy-induced reactions, valued for their high tissue penetration and clinical relevance (Scheme 1a).^4−7^ Essentially, reactive particles generated by radiolysis, such as hydroxyl radical (·OH), hydrogen radical (H·), and hydrated electron (e^–^_aq_),^8,9^ facilitate the release of antitumor agents from small molecules,^10,11^ antibody–drug conjugates,^12,13^ and nanomaterials,^14^ enabling the combined treatment to suppress primary tumors with minimal systemic toxicity.^15−17^ However, few tumor cells could migrate from primary tumor and accumulate in distant organs,^18^ leading to metastasis, which accounts for over 90% of cancer deaths and presents a major challenge to conventional therapies.^19,20^ Thus, expanding the prodrug activation strategy for metastasis is crucial, yet challenging.

Targeted radionuclide therapy, a treatment modality delivering radioactive atoms to tumor-associating targets, is emerging as a promising approach against metastasis due to its efficacy and safety.^21−23^ With the growing interest in targeted radionuclide therapy for cancer treatment, over 50 radiopharmaceuticals have gained approval by the U.S. Food and Drug Administration (FDA) in recent decades.^24^ Notably, [^177^Lu]Lu-vipivotide tetraxetan (Pluvicto), a recent FDA-approved radiopharmaceutical for the treatment of metastatic castration-resistant prostate cancer, generated $980 million in sales in its first full year on the market.^25^ This success underscores the increasing need for targeted radionuclide therapy for its clinical benefits in cancer treatment. Thus, exploiting the chemical reactivity of radionuclides and developing their combination therapeutic modalities will be highly important for their future clinical applications. With the growing clinical demand for targeted radionuclide therapy, advancing its integration with other therapies to enhance its application in earlier lines of treatment and for additional indications holds significant clinical value.^26^

Pt(IV) prodrugs have drawn attention as a kinetically inert form of Pt(II) drugs, minimizing “off-target” side effects and enhancing biocompatibility.^27−30^ Despite considerable efforts to promote the clinical translation of Pt(IV) prodrugs, none have been approved yet.^31^ Recently, we have reported a radiotherapy-activated platinum(IV) strategy with high clinical relevance for radiochemotherapy,^32^ facilitating the advancement of a radiotherapy-activated prodrug strategy toward clinical translation. Nevertheless, it remains ineffective in the treatment of metastatic tumors. Hence, in this work, we aim to leverage radiopharmaceuticals to expand the potential of the radiotherapy-activated prodrug strategy in the treatment of metastatic tumors while exploring the connection between the two strategies. Additionally, we intend to evaluate the therapeutic efficacy of combining radiopharmaceuticals with chemotherapeutic agents, which could provide valuable insights for future radiopharmaceutical therapies (Scheme 1b).

## Results and Discussion

### Internal Radiation-Induced Pt(IV) Complex Reduction in Aqueous Solution

**Figure 1:**
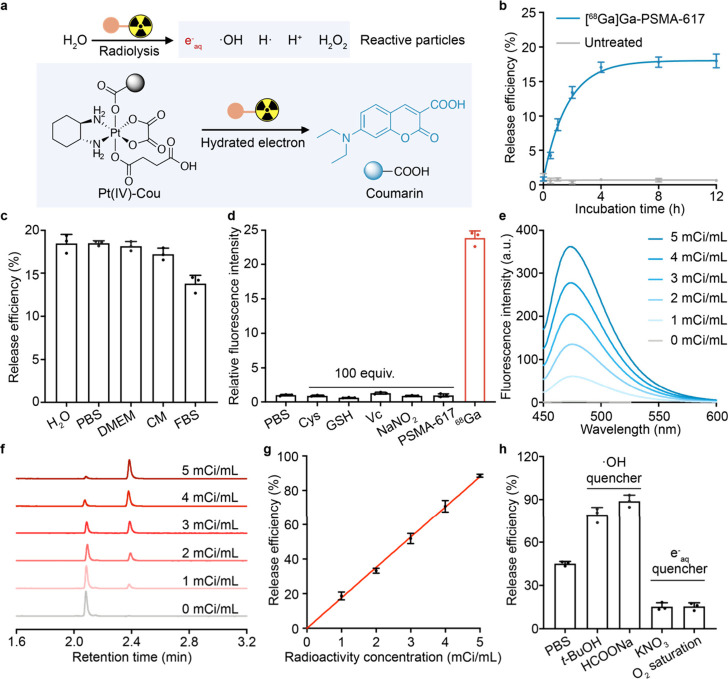
Radionuclide-induced cleavage of platinum(IV) complex axis ligands enables a radioactivity-dependent controlled release of fluorophore. (a) Schematic representation of radionuclide-induced release of 7-(diethylamino)coumarin-3-carboxylic acid (Coumarin) from Pt(IV)-Cou as a model reaction. (b) The release efficiency of coumarin from Pt(IV)-Cou incubated with 1 mCi/mL [^68^Ga]Ga-PSMA-617 after different times (*n* = 3 independent experiments). (c) Liquid chromatography assay of radionuclide-induced ligand release efficiency of platinum(IV) complex in various aqueous. DMEM, Dulbecco’s modified Eagle medium; CM, complete medium; FBS, fetal bovine serum. (d) Fluorescence emission assay (λ_ex_ = 432 nm and λ_em_ = 473 nm) of the stability of Pt(IV)-coumarin treated by various biologically relevant molecules compared with 1 mCi/mL [^68^Ga]Ga-PSMA-617 (^68^Ga) incubation (*n* = 3 independent experiments). (e) The fluorescence emission spectra (λ_ex_ = 432 nm), (f) UPLC-UV (λ_abs_ = 432 nm), and (g) efficacy of released coumarin from Pt(IV)-Cou after a radioactivity concentration gradient incubation of [^68^Ga]Ga-PSMA-617 in PBS for 12 h at room temperature. (h) The release efficiency of coumarin from Pt(IV)-Cou (10 μM in PBS) after a 12 h incubation with 3 mCi/mL [^68^Ga]Ga-PSMA-617 treated with e^–^_aq_ quencher (KNO_3_ and O_2_) and ·OH quencher (*t*-BuOH, HCOONa), respectively (*n* = 3 independent experiments).

To achieve our goal, we initially synthesized a Pt(IV) compound based on oxaliplatin and conjugated it with 7-(diethylamino)coumarin-3-carboxylic acid to form Pt(IV)-Cou as a model compound (Figure 1a; see the Supporting Information for details). Fluorescence of coumarin can be quenched by conjugated Pt due to the “heavy atom” effect, and once it is released from Pt(IV)-Cou, the fluorescence can be restored.^33^ Then, we incubated it with Gallium-68-labeled PSMA-617 solution (1 mCi/mL), an FDA-approved therapeutical radioligand, at a concentration of 10 μM. Compared to the untreated group, the fluorescence of the system exhibited a time-dependent increase with the introduction of Gallium-68 (^68^Ga) and peaked after 4 h (Figure 1b). It could be attributed to the fact that the half-life of ^68^Ga is only 68 min, with less than 10% of the radionuclide remaining in the system after 4 h. To further explore the feasibility of this reaction in a biological environment, we performed the experiment in various physiological solutions under the same conditions. As shown in Figure 1c, approximately 20% coumarin was released from the Pt(IV)-Cou in water and phosphate buffer saline (PBS) after a 12 h incubation with [^68^Ga]Ga-PSMA-617. The presence of these physiological solutions did not significantly alter the coumarin release efficiency. Additionally, Pt(IV)-Cou remained stable when exposed to biologically relevant reductants (1 mM), such as glutathione (GSH), vitamin C (Vc), and cysteine (Cys), for 24 h. Meanwhile, a 23-fold increase in fluorescence intensity was observed in the [^68^Ga]Ga-PSMA-617 group (Figure 1d). To better illustrate the feasibility of the strategy in living systems, we investigate the release efficiency of the reaction under various O_2_ concentrations. As shown in Figure S1, the reaction efficiency remained above 50% of its original value at oxygen concentrations ranging from 2 to 32 mmHg, which corresponds to the oxygen levels typically found in tumors. Together, these results indicate the occurrence of radionuclide-induced axis ligand release and its potential to operate in the living system.

Subsequently, we incubated Pt(IV)-Cou with [^68^Ga]Ga-PSMA-617 at a radioactivity concentration of 0–5 mCi/mL for 12 h to explore whether the reaction was radioactivity dependent. According to Figure 1e, the fluorescence intensity of the released coumarin exhibited an increase in a radioactivity-dependent manner. Quantitative analysis of the coumarin revealed a linear correlation with the gradient radioactivity concentration of ^68^Ga determined by the UPLC-UV assay (λ_abs_ = 432 nm, Figure 1f,g). Similar to coumarin, oxaliplatin generated by the reduction of Pt(IV)-Cou shows the same correlation with the radioactivity concentration of ^68^Ga (Figure S2a,b). Taken together, the results indicated that, similar to the external radiation-induced reaction, this radionuclide-induced axis release reaction is dose dependent and has the potential to be adapted to a controlled release system. To further investigate the connection between the reaction and the hydrated electron, we conducted the reaction in 10 mM solutions of *t*-BuOH, HCOONa, and KNO_3_, respectively. As depicted in Figure 1h, the addition of *t*-BuOH or HCOONa, the hydroxyl radical quenchers,^34^ increased the yield of coumarin. Conversely, the release efficiency of the system decreased to about 15% of the control group in the presence of hydrated electron quencher, KNO_3_ or O_2_ (saturated). The observation suggested that the hydrated electron from radiolysis played a key role during the reaction rather than the decay of β-rays from the radionuclide itself.

### Medical Radionuclide-Induced Activation in an Absorbed Dose Dependent Manner

**Figure 2:**
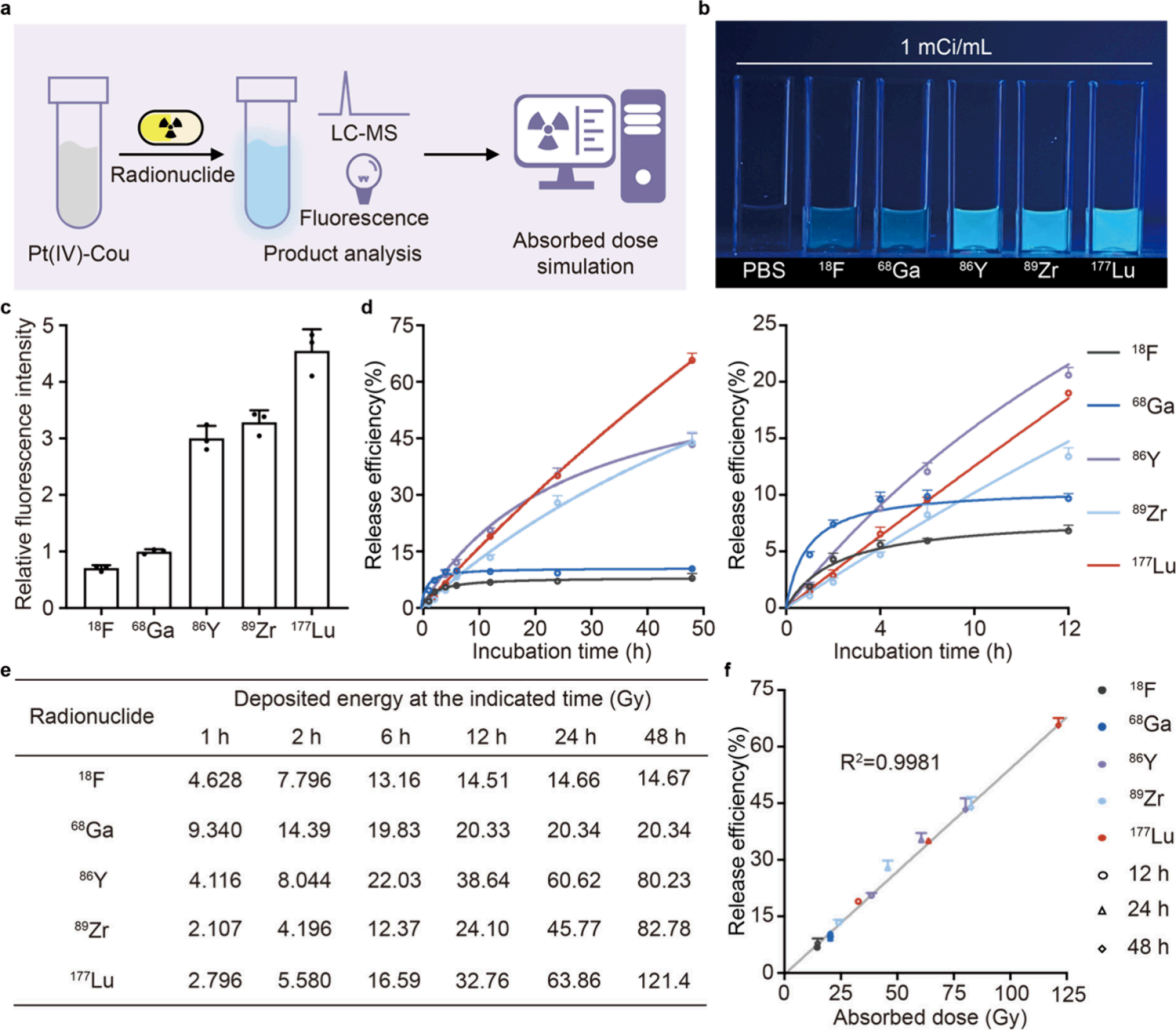
Various radionuclides could induce the release of axis ligands from Pt(IV)-Cou in an absorbed dose-dependent manner. (a) The workflow of computer-aided prediction of the release efficiency for the radionuclide-induced reaction. (b) Photograph and (c) fluorescence intensity (λ_ex_ = 432 nm and λ_em_ = 473 nm) of released coumarin from Pt(IV)-Cou (20 μM in PBS, pH 7.4) after a 48 h incubation with the indicated radionuclides (*n* = 3 independent experiments). Metal radionuclides were labeled with PSMA-617 while Fluorine-18 was converted to [^18^F]fludeoxyglucose to maintain the stability of the five tested radionuclides in PBS (pH = 7.4) during the experiment. (d) Release efficiency of coumarin from Pt(IV)-Cou incubated with various radionuclides after different times (*n* = 3 independent experiments). (e) Energy deposit at the indicated time of tested radionuclides simulated by Geant4. (f) Linear correlation between the release efficiency of coumarin and the simulated absorbed dose.

Building on the success with ^68^Ga, we sought to discover whether other medical radionuclides could undergo a similar reaction, which would be an important implication for further applications of the reaction. Thus, we conducted the incubation experiment using Fluorine-18, Gallium-68, Yttrium-86, Zirconium-89, and Lutetium-177 (1 mCi/mL), which are widely used in clinical practice, to investigate their capacity to induce the coumarin release from Pt(IV)-Cou (Figure 2a). First, we labeled the metal radionuclides with PSMA-617 and converted Fluorine-18 to [^18^F]fludeoxyglucose to ensure these radionuclides remained homogeneously dispersed in the solution, preventing precipitation. Then, we chose 48 h as the time point for reaction monitoring as other radionuclides have a long half-life compared to ^68^Ga, such as Yttrium-86, which has a half-life of 14.6 h. All solutions with the addition of radionuclides exhibited enhanced fluorescence compared to the untreated group after 48 h of incubation (Figure 2b,c), indicating the occurrence of radiopharmaceutical-induced axis ligand release. However, the radioactivity was not the only factor that determined the reaction efficiency as the fluorescence intensity varied among the systems at the same dose.

To further explore the mechanism of the reaction, we next investigated the correlation between the coumarin release and time. As illustrated in Figure 2d, the yield and rate of coumarin release were strongly influenced by the radionuclide employed. It can be attributed to the distinct decay properties, including energy and half-life, associated with each radioisotope. For example, ^68^Ga, characterized by the highest decay energy but the shortest half-life (68 min) among the five tested radionuclides (Table S1), resulted in Pt(IV)-Cou releasing coumarin with the most rapid rate at the initial stage of the reaction. However, it was almost completed in 4 h (approximately 4 half-lives). Of note, Lutetium-177, an FDA-approved therapeutic radioisotope,^35^ exhibited a nearly 5-fold increase in coumarin release compared to ^68^Ga owing to its extended longest half-life, suggesting remarkable potential for ligand release.

By analyzing the half-life and decay energy of the radionuclide, we could simply anticipate the rate and completion time of the reaction. However, achieving precise predictions regarding the quantity of ligand release is crucial for making the system applicable to a controlled release system. Drawing inspiration from the resemblance between this radionuclide-induced reaction and radiotherapy-activated reaction, we systemically evaluated the correlation between radiation absorbed dose to solution and time for each of these radionuclides (1 mCi/mL) using Geant4, radiation dose assessment software. The simulation results demonstrated the energy deposition of each system is radionuclide and time dependent (Figure 2e and Table S2). It is noteworthy that 1 mCi/mL Lutetium-177 could deliver an approximately 60 Gy radiation dose in 24 h, leading to the release of about 60% of the axial ligands from the Pt(IV) complex. Furthermore, the release of coumarin demonstrated a linear correlation with the radiation dose (Figure 2f), suggesting the release of the axis ligand can be evaluated through the prediction of the radiation dose, highlighting the importance of an on-demand release system.

### Tumor-Targeted Fluoroprobe Activation *in Vivo*

**Figure 3:**
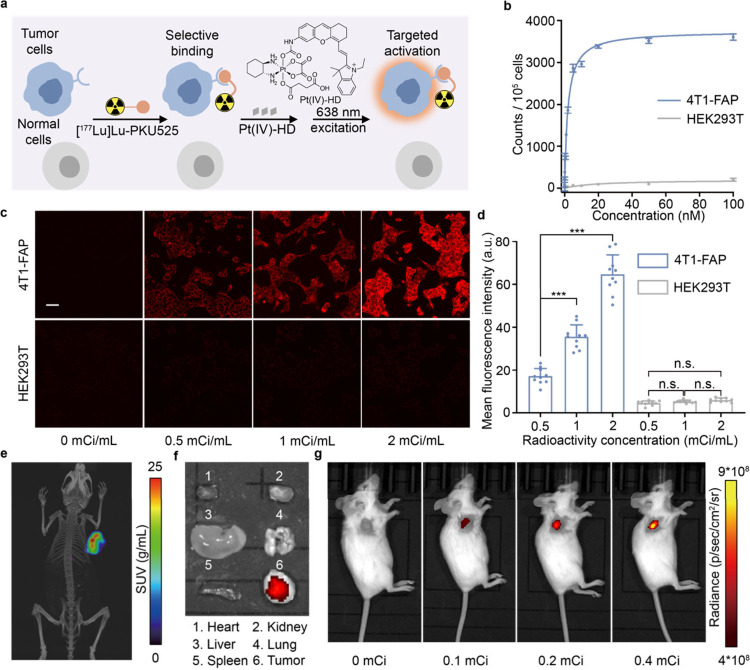
Targeted radionuclide therapy-induced activation of fluorophore *in vitro* and *in vivo* by the ligand cleavage of the Pt(IV) complex. (a) Schematic depicting the cell activation of Pt(IV)-HD using [^177^Lu]Lu-PKU525 in the presence of FAP on the cell membrane. (b) The cellular uptake of [^177^Lu]Lu-PKU525 in 4T1-FAP and HEK293T cells after incubation with a gradient concentration of [^177^Lu]Lu-PKU5255. (c) Representative confocal fluorescence images of 4T1-FAP and HEK293T cells after treatment with a radioactivity gradient of [^177^Lu]Lu-PKU525 (λ_ex_ = 638 nm). The cells were pretreated with different radioactivities of [^177^Lu]Lu-PKU525 under hypoxia conditions for 4 h followed by incubation with 10 μM Pt(IV)-HD. Scale bar, 25 μm. (d) Fluorescence intensity analysis of 4T1-FAP and HEK293T in the examined field of view by confocal microscopy (*n* = 10 fields of view). (e) Representative SPECT-CT image of 4T1-FAP tumor-bearing mice 7 days after administration of [^177^Lu]Lu-PKU525. (f) Representative major organ fluorescence image of 4T1-FAP tumor-bearing mice 12 h after the administration DPSE-encapsulated Pt(IV)-HD at 7-day postinjection of [^177^Lu]Lu-PKU525. (g) *In vivo* imaging of [^177^Lu]Lu-PKU525-induced fluorescence release in 4T1-FAP tumor-bearing mice. Data are shown as mean ± SD, one-way analysis of variance (ANOVA) followed by Tukey’s HSD post hoc test. n.s.: no significance; ***P < 0.001.

Given the tumor-targeting capability of radiopharmaceuticals, it is possible to achieve highly selective reactions within tissues. To account for this, a fluorogenic probe system, Pt(IV)-HD, was designed, wherein a near-infrared fluorescent hemicyanine dye is “caged” as a Pt(IV) complex by the carbamate (Figures 3a and S3a; see Supporting Information for details). Considering the efficiency of radionuclide-induced release as well as their therapeutic potential, we utilized Lutetium-177 for subsequent testing, as it exhibited the highest release efficiency and antitumor activity. We labeled it with PKU525, a humanized monoclonal antibody derived from sibrotuzumab, which is designed for fibroblast activation protein (FAP)-targeted radionuclide therapy, as FAP is ubiquitously expressed across various cancers.^36−38^ As shown in Figure 3b, the uptake of [^177^Lu]Lu-PKU525 by 4T1-FAP was significantly higher compared to that of HEK293T, a human embryonic kidney cell, indicating the high targeting ability of radiopharmaceuticals to tumor cells. Consequently, in the subsequent fluoroprobe-activation experiment, 4T1-FAP cells pretreated with [^177^Lu]Lu-PKU525 exhibited a more distinct fluorescence signal compared to HEK293T (Figure 3c). It is worth noting that the near-infrared dye in 4T1-FAP cells was released upon the addition of ^177^Lu in a positive correlative manner, with the mean fluorescence intensity in 4T1-FAP cells increasing to 17.1, 35.6, and 64.7 under the addition of 0.5 mCi/mL, 1 mCi/mL, and 2 mCi/mL [^177^Lu]Lu-PKU525, respectively. Similar experimental results were obtained in HT1080-FAP cells (Figure S3b,c). Conversely, almost no fluorescence was observed in HEK293T cells due to poor uptake (Figure 3d).

To further illustrate the tumor-targeted activation *in vivo*, we intravenously injected [^177^Lu]Lu-PKU525 to 4T1-FAP tumor-bearing mice with gradient radioactivity and observed its biodistribution by single photon emission computed tomography (SPECT-CT). As depicted in Figure 3e, [^177^Lu]Lu-PKU525 was notably enriched only at the tumor site 7 days postinjection. Then, we injected DSPE-PEG-encapsulated Pt(IV)-HD via tail-vein injection and collected the major organs and tumors after 24 h. Owing to the high tissue selectivity of [^177^Lu]Lu-PKU525, the fluorescence at the tumor site was remarkably higher than in other major organs (Figure 3f). Additionally, we observed a radioactivity-dependent fluorescence turn-on after the 12 h injection, indicating that the radiopharmaceutical-induced axis ligand release reaction remains highly selective and reactive *in vivo* (Figure 3g).

### Targeted Radionuclides Therapy-Activated Pt(IV) Twin Drugs Platform for Treatment of Metastasis

**Figure 4:**
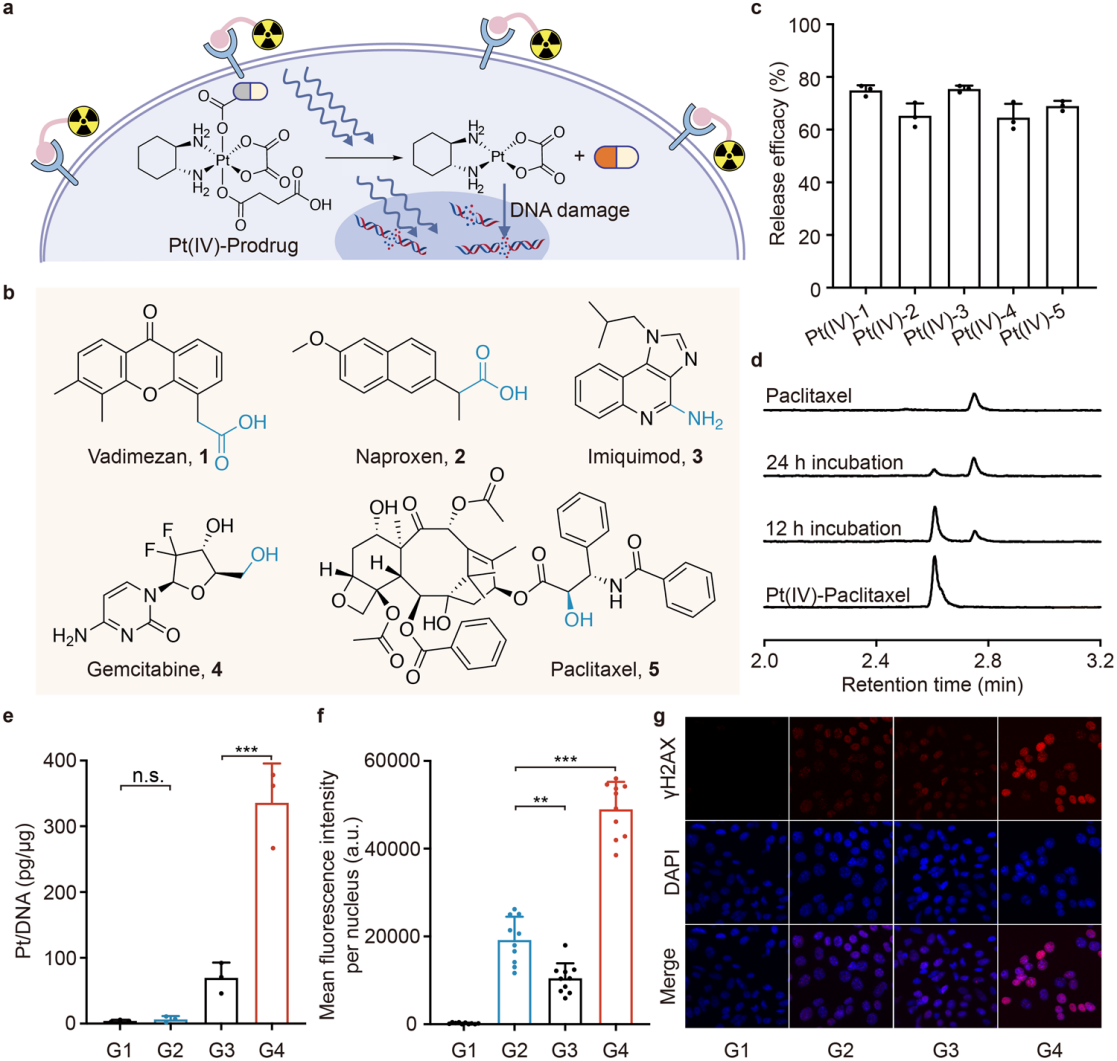
Targeted radionuclide therapy-induced reaction triggered the controlled release of clinically relevant drugs *in vitro*. (a) Schematic illustration of the controlled release of the oxaliplatin(IV) prodrug with the incubation of [^177^Lu]Lu-PKU525. Under the presence of the radiopharmaceutical, the oxaliplatin(IV) prodrug could be reduced to oxaliplatin with the simultaneous release of the antitumor drug, which together with the radiopharmaceuticals could cause DNA damage. (b) Chemical structures of drugs that show potential linkage sites. (c) The release efficiencies of corresponding Pt(IV)-prodrugs (10 μM in PBS) after 24 h of [^177^Lu]Lu-PKU525 incubation (*n* = 3 independent experiments). (d) The UPLC-UV (λ_abs_ = 238 nm) analysis of Pt(IV)-Paclitaxel, the reaction mixture of Pt(IV)-Paclitaxel after 12- and 24 h incubation with [^177^Lu]Lu-PKU525 and Paclitaxel, respectively. The concentration of the indicated substrates is 10 μM. (e) Pt levels in genomic DNA of 4T1-FAP cells after 24 h of incubation with Pt(IV)-Gem (10 μM) and [^177^Lu]Lu-PKU525 (0.1 mCi) or not (*n* = 4 independent experiments). (f) Quantitative fluorescence and (g) representative immunofluorescence images of γH2AX foci in 4T1-FAP cells (*n* = 10 independent fields of view). Nuclei were stained with DAPI (blue) and antibody to γH2AX (red). Scale bar, 25 μm. G1: PBS; G2: [^177^Lu]Lu-PKU525; G3: Pt(IV)-Gem; G4: [^177^Lu]Lu-PKU525 + Pt(IV)-Gem. Data are shown as mean ± SD, one-way analysis of variance (ANOVA) followed by Tukey’s HSD post hoc test, n.s., no significance, **P = 0.0019, ***P < 0.001.

With the success of this reaction in living systems, we sought to integrate it in the prodrug strategy, establishing a synergistic approach for the combination of chemotherapy and radiopharmaceutical therapy (Figure 4a). Thus, five drugs with various functional groups were conjugated with oxaliplatin(IV) complex to formulate Pt(IV) prodrug, named Pt(IV)-Vadimenzan (Pt(IV)-1), Pt(IV)-Naproxen (Pt(IV)-2), Pt(IV)-Imiquimod (Pt(IV)-3), Pt(IV)-Gemcitabine (Pt(IV)-4), and Pt(IV)-Paclitaxel (Pt(IV)-5) (Figure 4b; see the Supporting Information for details). Under the presence of [^177^Lu]Lu-PKU525, all of the Pt(IV) prodrugs could release their corresponding parent drug by the radionuclide-induced axis ligand with high efficiency in 24 h (Figure 4c). The UPLC-UV assay showed the sustained release of Paclitaxel from Pt(IV)-Paclitaxel during incubation (Figure 4d).

Notably, Pt(IV)-gemcitabine (Pt(IV)-Gem), whose active site was blocked with a Pt(IV) complex,^39^ exhibited a significant increase of the half maximal inhibitory concentration (IC_50_) from 1.87 ± 1.39 μM of oxaliplatin and 0.0741 ± 0.0386 μM of gemcitabine to 10.9 ± 3.85 μM against 4T1-FAP cells (Figure S4). It is well-known that cisplatin complexes can bind DNA bases, forming Pt-DNA adducts that cause DNA double-strand damage. In the 4T1-FAP cells treated with Pt(IV)-Gem, the Pt level in DNA increased from 4.40 ± 1.29 pg Pt per μg DNA to 335.8 ± 60.1 pg Pt per μg DNA after a 12 h incubation with [^177^Lu]Lu-PKU525 (Figure 4e). Furthermore, we performed a γH2AX staining assay to assess DNA damage in 4T1-FAP cells treated under different conditions (Figure 4f,g). No significant γH2AX fluorescence was observed in the PBS group, while the [^177^Lu]Lu-PKU525 and Pt(IV)-Gem groups exhibited moderate fluorescence. Notably, a 2.55-fold increase in fluorescence was observed in the Pt(IV)-Gem + [^177^Lu]Lu-PKU525 group compared to the [^177^Lu]Lu-PKU525 alone group. These findings indicated the release of oxaliplatin by the reduction of Pt(IV)-Gem, which subsequently caused DNA double-strand damage.

**Figure 5:**
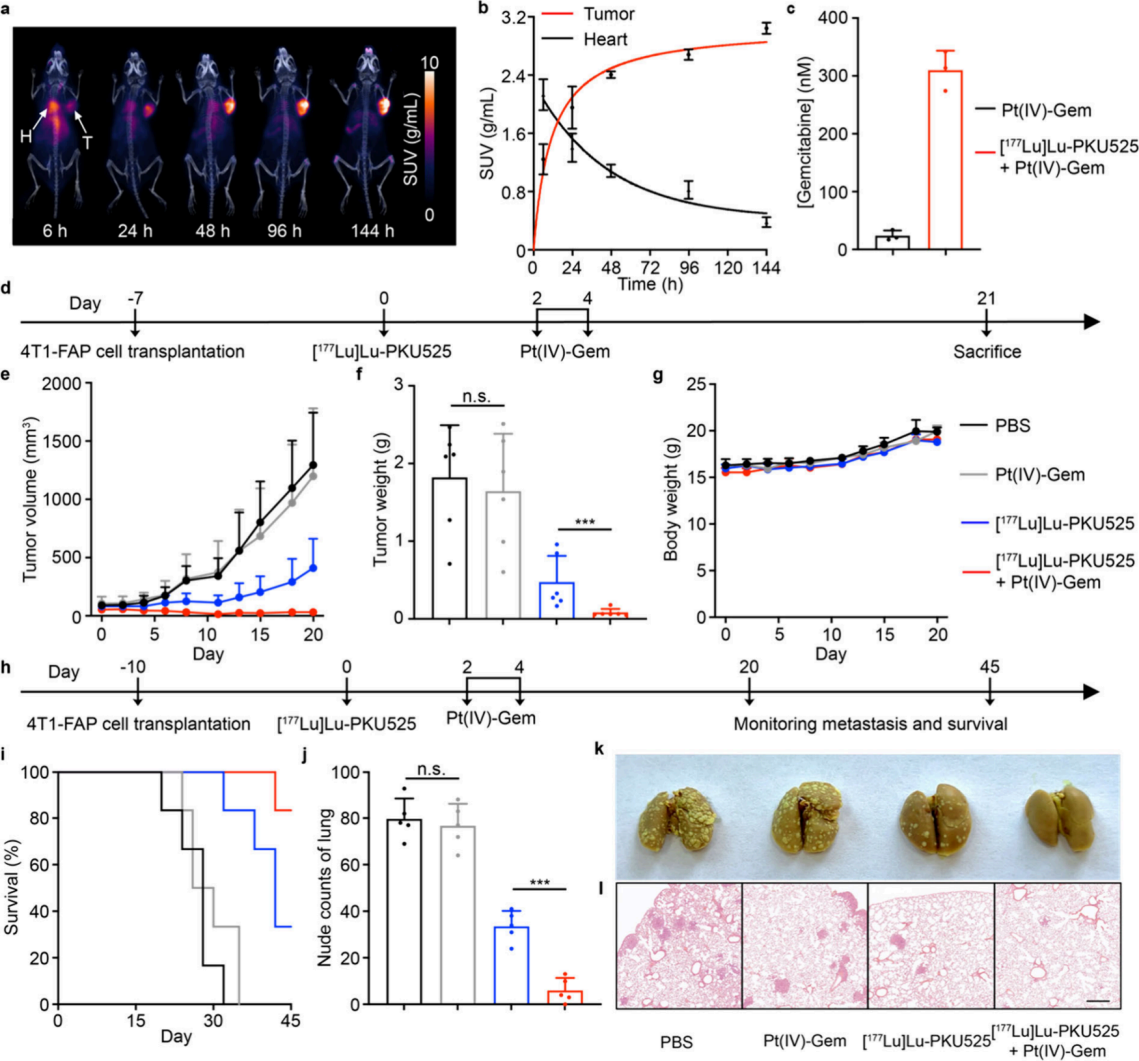
Targeted radionuclide therapy-induced controlled release of antitumor drug strategy suppressed the growth of subcutaneous tumors and lung metastasis in mice. (a) Dynamic PET-CT images of 4T1-FAP tumor-bearing mice at the indicated time after intravenous administration of [^89^Zr]Zr-PKU525. T, tumor; H, heart. (b) Time-activity curve of [^89^Zr]Zr-PKU525 in blood and tumor. (c) Evaluation of tumor concentration of Gemcitabine after mice were treated with Pt(IV)-Gem (20 mg/kg) or additionally with [^177^Lu]Lu-PKU525 (0.1 mCi) detected by UPLC-MS. (d) Treatment scheme of Pt(IV)-Gem (20 mg/kg) + [^177^Lu]Lu-PKU525 (0.1 mCi each mouse) in 4T1-FAP tumor-bearing mice. (e) Tumor growth and (f) tumor weight of tumor-bearing mice after the indicated treatments (*n* = 6 mice). (g) Record of body weight of the indicated treatment groups. (h) Schematic illustration of [^177^Lu]Lu-PKU525 + Pt(IV)-Gem inhibited the 4T1-FAP lung metastases tumor model. (i) Survival curve of mice during the treatment. (j) Quantification of metastatic lesions of the lung nodules at day 20 (*n* = 5 mice). (k) Representative photographs and (l) H&E to show the gross appearance of tumor nodules in the lungs. Scale bar = 400 μm. (c, f, j), Data are shown as mean ± SD, one-way analysis of variance (ANOVA) followed by Tukey’s HSD post hoc test. n.s.: no significance; ***P < 0.001.

In the pursuit of advancing the radiopharmaceutical-induced reduction system, we evaluated the therapeutic efficacy of the strategy on 4T1-FAP tumor-bearing mice. To determine the optimal timing and minimize side effects for the therapy, we initially investigated the pharmacokinetics of the antibody-radionuclide conjugate using nuclear imaging techniques including positron emission tomography–computed tomography (PET-CT) and SPECT-CT. As shown in Figure 5a, PET-CT imaging of Zirconium-89 labeled PKU525 demonstrated that the radiopharmaceutical held good tumor-targeting ability and could remain in the tumor for more than 144 h, which implied we could regulate the timing of Pt(IV)-Gem administration to achieve prodrug activation predominantly in the tumor site rather than in major organs. By analyzing the time-activity curve (TAC) of [^89^Zr]Zr-PKU525 in the tumor and heart (Figure 5b), we found that the standard uptake value (SUV) of the tumor could be maintained over 2.41 after 48 h of the administration of PKU525, while that in the blood decreased to 1.08. This suggested that the tumor-to-blood ratio could be maintained above 2.23 for an extended period after that time point. Similar results were found in the SPECT-CT of [^177^Lu]Lu-PKU525 (Figure S5a,b), leading us to select 48 h postinjection of PKU525 as the optimal time point for the administration of Pt(IV)-Gem.

Additionally, we examined the pharmacokinetics of Pt(IV)-Gem following the assessment of PKU525. In 4T1-FAP tumor-bearing mice, 20 mg/kg Pt(IV)-Gem was administered, and the mice were sacrificed at specified time intervals. ICP-MS analysis was performed on digested samples from major organs to determine the platinum concentrations. The tumor uptake of Pt(IV)-Gem peaked at approximately 2 h after the injection and declined subsequently, resulting in a final concentration at about 2 μM at 48 h postinjection (Figure S6). The unabsorbed prodrug was rapidly eliminated from the bloodstream and exerted by the metabolic system after 48 h. Afterward, we evaluated the release of gemcitabine to optimize the dosing regimen. Mice were treated with Pt(IV)-Gem 2 days after the injection of [^177^Lu]Lu-PKU525 and sacrificed 48 h later, as the majority of prodrug was metabolized by the tumor at that time. Tumors were harvested, and the drug concentration was tested by UPLC-MS. As depicted in Figure 5c, the amount of gemcitabine in the tumor after combination therapy was increased from 24.0 ± 9.0 to 310.1 ± 33.9 nM, which is higher than the IC_50_, suggesting the targeted radionuclide therapy-induced drug release *in vivo* is conducive to achieving effective tumor outcomes and extending survival.

For the tumor therapy studies, 4T1-FAP tumor-bearing mice were randomly divided into 4 groups, including those treated with PBS, Pt(IV)-Gem, [^177^Lu]Lu-PKU525, and Pt(IV)-Gem + [^177^Lu]Lu-PKU525 (*n* = 6 mice), initiated when the tumor volume reached approximately 50 mm^3^. In the groups receiving radiopharmaceutical administration, 0.1 mCi of [^177^Lu]Lu-PKU525 was injected per mouse on Day 0. On Days 2 and 4, 20 mg/kg Pt(IV)-Gem was given to initiate the combination therapy (Figure 5d). After 21 days, a remarkable tumor suppression was observed in the Pt(IV)-Gem + [^177^Lu]Lu-PKU525 group, in which tumor volume was about 31.3 ± 22.1 mm^3^ on that day, while the tumor size of the PBS group and [^177^Lu]Lu-PKU525 reached up to 1296 ± 447.3 mm^3^ and 411.6 ± 581.1 mm^3^, respectively. No significant tumor suppression was observed when mice were treated with Pt(IV)-Gem alone during the experiment (Figure 5e). The tumor weight measurements and photographs confirmed the antitumor effect resulting from the radiopharmaceutical-induced release of oxaliplatin and gemcitabine (Figure 5f and Figure S7a,b). It is worth noting that mice treated with Pt(IV) and [^177^Lu]Lu-PKU525 did not exhibit significant changes in body weight during the treatment. Biosafety was further assessed by blood routine analysis and H&E staining, with the results revealing no abnormalities in blood parameters or major organs (Figures S8 and S9). Collectively, these findings indicated the satisfactory biosafety of the targeted radionuclide therapy-activated prodrug strategy.

Given the advantages of targeted radionuclide therapy in treating metastatic tumors compared to traditional radiotherapy, we proceeded to investigate the antimetastatic effect in the 4T1-FAP lung metastatic tumor model by using the combination therapy approach. As illustrated in Figure 5h, 4T1-FAP cells were intravenously injected to the female Balb/c mice. After 10 days, the mice were randomly divided into 4 groups (*n* = 6 mice) and received the same treatment procedure as mentioned above. Five out of the 6 mice in the Pt(IV)-Gem and [^177^Lu]Lu-PKU525 group survived for more than 45 days, while no mice in PBS or the Pt(IV)-Gem alone group survived beyond 35 days (Figure 5i). The [^177^Lu]Lu-PKU525 alone group exhibited limited metastasis treatment ability, with over half of the mice in the group succumbing within 45 days. For a more comprehensive evaluation of the lung metastasis, lung tissue from all groups was collected on day 20. Similarly, the combination of Pt(IV)-Gem and [^177^Lu]Lu-PKU525 exerted the potential to prevent metastasis, reducing the average number of lung nodules to 6.0 from 79.8 in the PBS group (Figure 5j,k). Moreover, from the gross appearance of lung tissue and corresponding H&E staining shown in Figure 5l, large areas of metastatic lesions appeared on the surface of lungs except the Pt(IV)-Gem + [^177^Lu]Lu-PKU525 group, implying the therapeutic capability of this strategy for metastatic tumors.

## Conclusion

In summary, we developed a targeted radionuclide therapy-induced reduction system to generate oxaliplatin and the antitumor agent simultaneously via the release of axis ligands from Pt(IV) complexes. Of note, we observed that various radionuclides could induce the release with different characterizations. Among these, ^68^Ga shows the fastest reaction rate, while Lutetium-177 induces the most ligand release under the same radioactivity concentration, which can be attributed to the difference in energy deposited induced by the radioisotopes. With the help of computational simulation, we revealed the linear correlation between the release of ligands and absorbed dose caused by radionuclides, which is crucial to the selection of reactive radionuclides and the assessment of prodrug release in the future. Meanwhile, the success of this radiotherapy-inspired reaction implies the previously reported radiation-responsive groups could respond to radionuclides as well.

Benefiting from the advantages of targeted radionuclide therapy, the release of ligands is highly tumor-targeted and minimizes the side effects to the surrounding normal organs, as the low energy β-ray emitted by Lutetium-177, for instance, could only penetrate approximately 0.6 mm in tissue. The combination of Pt(IV)-Gemcitabine and ^177^Lu labeled radiopharmaceutical substantially suppressed subcutaneous tumor growth in mice compared to their administration alone and had no significant adverse effects. It is worth noting that this strategy works on the lung-metastatic model as well. With this work, we hope to fill the gap of the radiation-induced reaction on β-emitting radionuclides and develop more radiopharmaceutical-activatable platinum(IV) prodrugs, such as carboplatin and nedaplatin, to promote the strategy in accommodating more cancer treatment.

## Supporting Information Available

Reagents and apparatus, chemical synthesis, experimental details, supplementary figures, and NMR spectra and high-resolution mass spectra for all compounds (PDF)
