# Supplementary material for: Targeted Radionuclide Therapy Activates Prodrugs for Treating Metastasis
Source: ACS Cent Sci. 2024 Dec 5;10(12):2321–30. doi: 10.1021/acscentsci.4c01369 (PMC11672548; doi:10.1021/acscentsci.4c01369)
Supplement: Supplementary file 1 — oc4c01369_si_001.pdf [file oc4c01369_si_001.pdf]

Supporting Information for

Targeted Radionuclide Therapy Activates Prodrugs for Treating Metastasis

Zhibin Guo[1], Xuanyu Wang[1], Yi Han[2], Siyong Shen[1], Peng Tian[1], Yuchen Hu[1], Zexuan Ding[3], Qunfeng Fu[1], Zhibo Liu[1,3-5*]

[1]Beijing National Laboratory for Molecular Sciences, Radiochemistry and Radiation Chemistry Key Laboratory of Fundamental Science, College of Chemistry and Molecular Engineering, Peking University, Beijing 100871, China

[2]China Institute of Atomic Energy, Institute of Nuclear Technology, Beijing 102413, China

[3]Changping Laboratory, Beijing 102206, China

[4]Peking University-Tsinghua University Centre for Life Sciences, Peking University, Beijing 100871, China

[5]Key Laboratory of Carcinogenesis and Translational Research (Ministry of Education/Beijing), NMPA Key Laboratory for Research and Evaluation of Radiopharmaceuticals (National Medical Products Administration), Department of Nuclear Medicine, Peking University Cancer Hospital & Institute, Beijing 100142, China

*Corresponding author, email: zbliu@pku.edu.cn

Table of Content

Reagents and Apparatus 1
Chemical Synthesis 2
Labeling Method 5
Deoxygenation Method 6
Dose Simulation 6
Biological Methods 6
Cell Culture 6
Cell viability assays 6
Cellular uptake of ^{177}Lu-PKU525 6
Confocal imaging 6
Immunofluorescence assay for cellar DNA injury 7
Measurement of oxaliplatin concentrations in cells 7
Animal model 7
Fluorescence image of mice 7
PET image of mice 8
SPECT image of mice 8
Biodistribution of Pt 8
Treatment of mice 8
H&E staining 8
Reference 8
Supplementary Tables 10
Supplementary Figures 12

Reagents and Apparatus

All chemical reagents, purchased from BidePharm (China), Energy Chemical (China), J&K (China), Inno-chem (China), and Sinopharm (China), were used as received without further purification. Ultrapure water (18.2 MΩ·cm) employed throughout was from a Milli-Q reference system (Millipore). ^{18}F-fluorodeoxyglucose ([^{18}F]FDG) was purchased from HTA Co. Ltd. $^{68}GaCl_3$ was eluted with a solution of 0.6 M hydrochloride from a ^{68}Ge–^{68}Ga generator (iThemba LABS). $^{86}YCl_3$ was produced on the CYCIAE-14 Cyclotron at Peking University by ^{86}Sr(p, n)^{86}Y; ^{89}Zr was produced on the CYCIAE-14 Cyclotron at Peking University by the ^{89}Y(p, n)^{89}Zr reaction and purified to yield ^{89}Zr in 0.1 M oxalic acid. ^{177}Lu (37 GBq/mL in 0.1 M HCl) was purchased from ITG (Germany). Cell Counting Kit-8 (CCK-8) was purchased from Biyuntian Biotechnology Institute. Anti-γ H2A.X (phospho S139) Goat Anti-Rabbit IgG H&L (Alexa Fluor® 647) and FITC-labeled anti-α-tubulin antibody were purchased from Abcam. The anti-FAP antibody PKU525 was produced by WuXi Biologics. DNA extraction kit DP304 (TIANGEM®) were used to extract genomic DNA.

Ultra-performance liquid chromatography-mass spectrometry (UPLC-MS) was performed on ACQUITY UPLC H-Class PLUS instrument equipped with Waters PDA eλ Detector and a Waters SQ Detector 2 mass spectrometer. High-resolution mass spectroscopy was performed on a Bruker Fourier Transform Ion Cyclotron Resonance Mass Spectrometer. Nuclear magnetic resonance (NMR) spectra were recorded on Bruker AVANCE 400 MHz spectrometer and Bruker AVANCE 600 MHz spectrometer. Signals are presented as parts per million (ppm), and multiplicity is presented as single (s), broad (b), doublet (d), triplet (t), quartet (q), or multiplet (m). Monte Carlo Simulation used Geant4 software. Fluorescence spectra were measured on an F-7000 spectrophotometer (Hitachi, Japan). PKU525 concentration was measured by Nanodrop One (Thermo Fisher Scientific Inc.). Confocal fluorescence images were recorded on an A1R-si Laser Scanning Confocal Microscope (Nikon, Japan). In vivo optical images were taken on IVIS Lumina III in vivo imaging system. PET/CT images were taken on microPET (Mediso Medical Imaging Systems). SPECT/CT images were taken by lnliView-3000B PET/SPECT/CT imaging system. *In vivo* optical images were taken on IVIS Lumina III in vivo imaging system. ICP-MS (Inductively Coupled Plasma Mass Spectrometry) analysis was conducted on NexlON 350X ICP-MS and organs and tumor were digested with 68% HNO_3 (3 mL/1 g organs) using microwave accelerated reaction system (Mars, CEM) (140 W, 10 min).

Chemical Synthesis

Oxali-Pt(IV) was synthesized as Scheme S1 shown. Oxaliplatin (2.5 g) was mixed with 6 mL H_2O_2, and was added into 7.5 mL water. After stirring for 5 h under 50°C, the reaction completed. Afterwards, the solution was cooled down to room temperature and was centrifuged at 4000 rpm for 3 minutes. The precipitation was lyophilized to obtain the product with a yield of 90%.

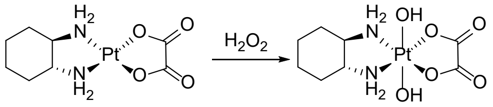

Scheme S1. Synthesis of Oxali-Pt(IV).

Pt(IV)-Coumarin was synthesized as Scheme S2 shown.

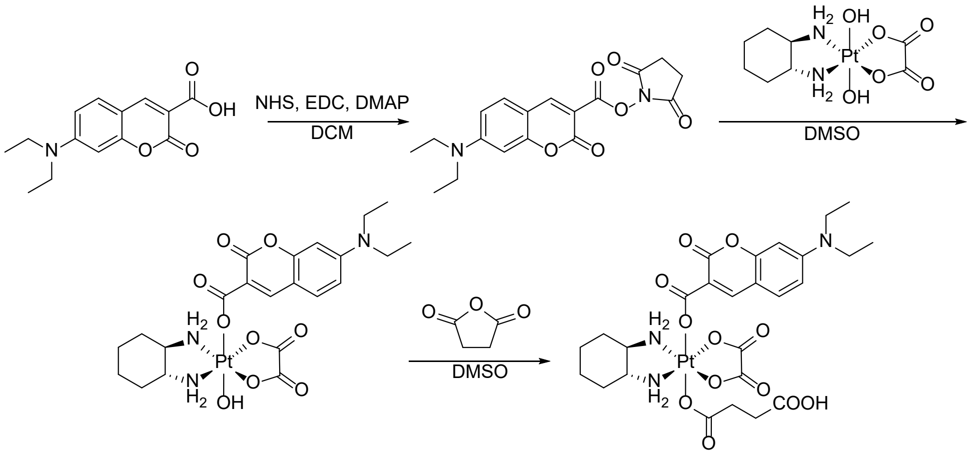

Scheme S2. Synthesis of Pt(IV)-Cou.

522 mg 7-(diethylamino)coumarin-3-carboxylic acid (1 equiv.), 276 mg NHS (1.2 equiv.), 460 mg EDCI (1.2 equiv.) and 485 mg Et_3N (2.4 equiv.) were dissolved in 10 mL DCM and stirred overnight at room temperature. After the reaction completed and the solvent was evaporated under reduced pressure, the system was washed twice with water and then the product was purified by column chromatography with a yield of 70%. The coumarin active ester synthesized in the above steps (240 mg, 1 equiv.) and Oxali-Pt(IV) (570 mg, 2 equiv.) were added to 2 mL anhydrous DMSO, stirred overnight at 60°C. Then 2 mL DCM was added. The system was precipitated with 20 mL anhydrous ether and centrifuged to obtain coumarin oxaliplatin. The obtained coumarin oxaliplatin (67 mg, 1 equiv.) and succinic anhydride (40 mg, 4 equiv.) were dissolved in 1 mL DMSO and reacted overnight. After the reaction completed, 1 mL DCM was added and the system was precipitated with 15 mL anhydrous ether. The crude product was centrifuged. Pt(IV)-Cou was obtained by using preparative HPLC for further purification, with a product of 60%.

The preparation methods of Pt(IV)-Vadimezan and Pt(IV)-Naproxen were consistent with that of Pt(IV)-Coumarin.

Pt(IV)-Cou was obtained as yellow compound (60%). ^{1}H NMR (400 MHz, DMSO-d_6) δ 8.37(s, 1H), 8.36 – 7.80 (m, 4H), 7.52 (d, J = 9.0 Hz, 1H), 6.74 (dd, J = 9.0, 2.4 Hz, 1H), 6.50 (d, J = 2.3 Hz, 1H), 3.46 (q, J = 7.0 Hz, 4H), 3.01 – 2.83 (m, 1H), 2.63 (s, 1H), 2.61 – 2.52 (m, 2H), 2.45 – 2.36 (m, 2H), 2.26 – 2.01 (m, 2H), 1.66 – 1.33 (m, 4H), 1.18 (d, J = 9.0 Hz, 2H), 1.13 (t, J = 7.0 Hz, 6H). ^{13}C NMR (151 MHz, DMSO-d_6) δ 180.25, 174.21, 170.30, 164.15, 158.16, 157.81, 152.69, 148.41, 131.60, 110.95, 110.03, 107.36, 96.32, 61.59, 60.93, 44.73, 31.42, 31.37, 31.04, 30.12, 24.12, 24.03, 12.81. HRMS (ESI$^+$): m/z calculated for $C_{26}H_{34}N_3O_{12}Pt^+$ ($[M+H]^+$): 775.1785, found: 775.1787

Pt(IV)-Vadimezan was obtained as white compound (50%). ^{1}H NMR (600 MHz, DMSO-d_6) δ 12.12 (s, 1H), 8.52 – 8.07 (m, 4H), 8.06 (dd, J = 7.9, 1.7 Hz, 1H), 7.90 (d, J = 8.1 Hz, 1H), 7.73 (dd, J = 7.3, 1.7 Hz, 1H), 7.37 (t, J = 7.6 Hz, 1H), 7.28 (d, J = 8.1 Hz, 1H), 4.02 (d, J = 7.9 Hz, 2H), 2.63 – 2.51 (m, 2H), 2.50 – 2.43 (m, 2H), 2.41 (s, 3H), 2.37 (s, 3H), 2.09 – 2.01 (m, 2H), 1.57 – 1.42 (m, 2H), 1.42 – 1.24 (m, 2H), 1.24 – 0.89 (m, 2H). ^{13}C NMR (151 MHz, DMSO-d_6) δ 180.08, 177.90, 176.75, 174.20, 163.77, 163.74, 154.09, 153.86, 144.95, 136.89, 126.39, 126.14, 125.91, 124.92, 123.97, 123.01, 121.07, 119.31, 61.42, 61.31, 37.09, 31.42, 31.33, 30.99, 30.04, 24.00, 23.88, 20.70, 11.95, 0.59. HRMS (ESI$^+$): m/z calculated for $C_{29}H_{33}N_2O_{12}Pt^+$ ([M+H]$^+$): 796.1676, found: 796.1674

Pt(IV)-Naproxen was obtained as white compound (54%).^{1}H NMR (600 MHz, DMSO-d_6) δ 12.11 (s, 1H), 8.32 (s, 2H), 8.23 – 7.92 (m, 2H), 7.76 (dd, J = 9.0, 2.0 Hz, 1H), 7.72 – 7.69 (m, 1H), 7.68 (d, J = 2.1 Hz, 1H), 7.37 (ddd, J = 8.2, 5.9, 1.9 Hz, 1H), 7.27 (d, J = 2.4 Hz, 1H), 7.13 (dt, J = 8.9, 2.4 Hz, 1H), 3.89 – 3.86 (m, 1H), 3.85 (s, 3H), 2.55 – 2.51 (m, 2H), 2.41 – 2.33 (m, 2H), 2.28 – 2.14 (m, 1H), 2.13 – 1.98 (m, 2H), 1.97 – 1.86 (m, 1H), 1.40 (dd, J = 7.2, 3.6 Hz, 3H), 1.36 – 0.94 (m, 6H). ^{13}C NMR (151 MHz, DMSO-d_6) δ 182.31, 180.13, 174.20, 163.80, 163.74, 157.53, 137.35, 133.58, 129.60, 128.83, 127.17, 127.08, 125.87, 119.02, 106.15, 61.57, 61.08, 55.64, 46.96, 31.45, 31.27, 31.01, 30.08, 23.87, 23.73, 19.41. HRMS (ESI$^+$): m/z calculated for $C_{26}H_{33}N_2O_{11}Pt^+$ ([M+H]$^+$): 744.1727, found: 744.1726.

Pt(IV)-HD was synthesized as Scheme S3 shown.

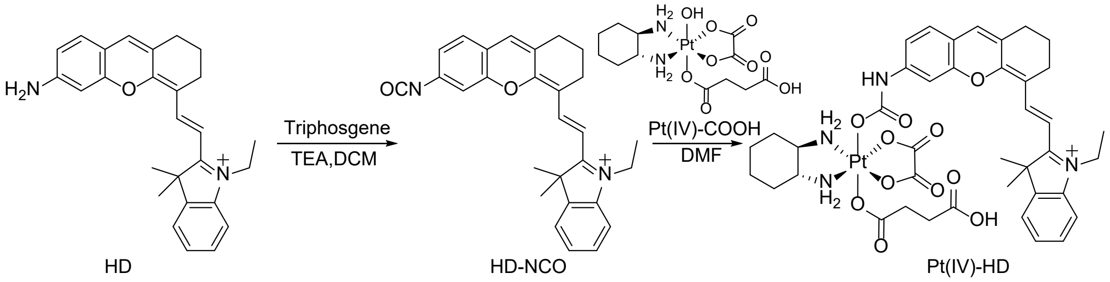

Scheme S3. Synthesis of Pt(IV)-HD.

Hemicyanine Dye (HD) was synthesized following the reported procedure[1]. 200 mg HD (1.0 equiv.) and 56 mg triphosgene (0.5 equiv.) were dissolve in 20 mL anhydrous DCM in ice Water Bath, under the N_2 atmosphere 155 μL TEA was added into the solution. When the reaction was completed, the solvent was evaporated under reduced pressure, following the addition of 220 mg Pt(IV)-COOH and 5 mL anhydrous DMF, which could be obtained following the reported procedure[2]. After the reaction completed, Pt(IV)-HD was obtained by using preparative HPLC for further purification, with a product of 60%.

Pt(IV)-HD was obtained as dark blue compound (47%). ^{1}H NMR (600 MHz, DMSO-d_6) δ 9.80 (s, 1H), 8.61 (d, J = 14.5 Hz, 1H), 8.51 (d, J = 6.1 Hz, 1H), 8.35 (s, 1H), 8.11 (d, J = 5.7 Hz, 1H), 7.76 (d, J = 7.4 Hz, 1H), 7.70 – 7.68 (m, 1H), 7.56 (s, 1H), 7.51 – 7.47 (m, 2H), 6.61 – 6.59 (m, 1H), 4.45 (q, J = 7.2 Hz, 2H), 2.72 (dt, J = 24.7, 5.9 Hz, 4H), 2.56 (s, 2H), 2.43 – 2.37 (m, 2H), 2.19 (s, 2H), 1.77 (d, J = 6.7 Hz, 6H), 1.61 – 1.48 (m, 4H), 1.40 (t, J = 7.1 Hz, 3H), 1.33 – 1.09 (m, 4H), 1.03 – 0.94 (m, 2H).. HRMS (ESI$^+$): m/z calculated for $C_{40}H_{47}N_4O_{12}Pt^+$ ([M+H]$^+$): 954.2884, found: 954.2874.

Pt(IV)-Gemcitabine (Pt(IV)-Gem) was synthesized as Scheme S4 shown.

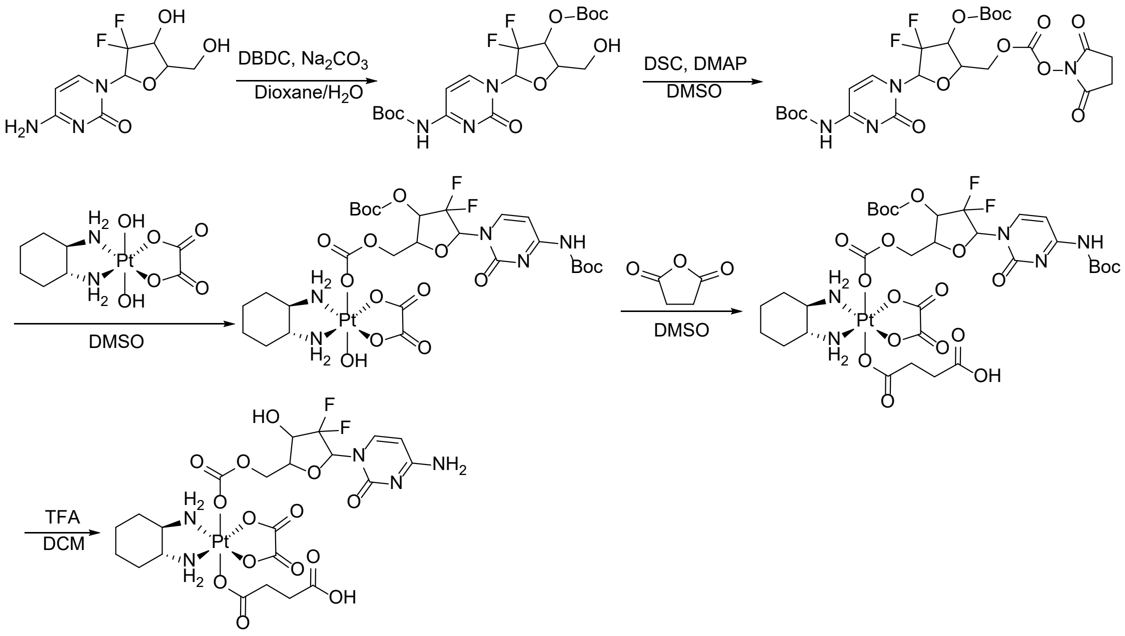

Scheme S4. General synthetic route of Pt(IV)-Gem.

Gemcitabine (526 mg, 1 equiv.) and Na_2CO_3 (1.06 g, 5 equiv.) were added to 4 mL 1,4-dioxane and 1 mL of water, followed by addition of DBDC (440 mg, 1 equiv.). Stirred the mixture at 24°C for 48 hours. After adding 2 mL of water, the solution was extracted with 2 × 30mL of ethyl acetate, washed with water, and dried by anhydrous Na_2SO_4. The organic solvent was concentrated under reduced pressure. Afterwards, the product was separated using column chromatography with a yield of 80%.

Boc-protected gemcitabine (100 mg, 1 equiv.), DSC (83 mg, 1.5 equiv.), and DMAP (2.5 mg, 0.1 equiv.) were dissolved in 1 mL anhydrous DMSO. After the complete conversion of the raw material, Oxali-Pt(IV) (80 mg, 1.1 equivalent) was added, reacting overnight at 40°C. Then added 1 mL DCM and precipitated the system with 15 mL anhydrous ether. Boc-protected gemcitabine oxaliplatin was obtained by centrifugation in a yield of 55%.

Boc-protected gemcitabine oxaliplatin (80 mg, 1 equiv.) and succinic anhydride (35 mg, 4 equiv.) were dissolved in 1 mL DMSO and reacted overnight. Followed by the addition of 1 mL DCM, the system was precipitated with 15 mL anhydrous ether and centrifuged to obtain the crude product. The obtained crude product was dissolved in 2 mL DCM and 2 mL trifluoroacetic acid was added. After 1 hour of reaction, the solvent was evaporated under reduced pressure. Preparative HPLC was used for further purification to obtain Pt(IV)-Gem with a yield of 49%.

The preparation methods of Pt(IV)-Imiquimod and Pt(IV)-Paclitaxel were consistent with those of Pt(IV)-Gem.

Pt(IV)-Imiquimod was obtained as white compound (62%). ^1H NMR (400 MHz, DMSO-d_6) δ 8.96 (s, 1H), 8.80 – 8.31 (m, 4H), 8.24 (s, 1H), 8.19 (d, J = 8.1 Hz, 1H), 7.94 (d, J = 8.2 Hz, 1H), 7.62 (t, J = 7.6 Hz, 1H), 7.56 (d, J = 7.7 Hz, 1H), 4.47 (d, J = 7.4 Hz, 2H), 2.85 – 2.62 (m, 2H), 2.59 – 2.50 (m, 2H), 2.45 – 2.35 (m, 2H), 2.25 – 2.16 (m, 1H), 2.16 – 2.11 (m, 2H), 1.56 – 1.40 (m, 4H), 1.23 – 1.13 (m, 2H), 0.93 (d, J = 4.1 Hz, 6H).^{13}C NMR (151 MHz, DMSO-d_6) δ 180.11, 174.31, 164.16, 164.06, 159.10, 144.98, 133.27, 130.53, 129.61, 127.70, 125.16, 121.16, 121.02, 116.77, 114.05, 61.56, 61.20, 54.00, 31.45, 31.38, 31.01, 30.22, 28.90, 24.11, 23.96, 19.82, 19.80. HRMS (ESI$^+$): m/z calculated for $C_{27}H_{35}N_6O_{10}Pt^+$ ([M+H]$^+$): 798.2057, found: 798.2053.

Pt(IV)-Gemcitabine (Pt(IV)-Gem) was obtained as white compound (49%). ^1H NMR (600 MHz, $CDCl_3$)

δ 8.71 – 7.73 (m, 4H), 7.50 (d, J = 7.5 Hz, 1H), 7.40 (d, 2H), 6.42 (s, 1H), 6.15 (s, 1H), 5.79 (d, J = 7.5 Hz, 1H), 4.29 (dd, J = 12.3, 2.4 Hz, 1H), 4.17 (dd, J = 12.2, 7.1 Hz, 1H), 4.10 (m, 1H), 3.97 (ddd, J = 8.4, 7.2, 2.5 Hz,1H), 2.69 – 2.56 (m, 2H), 2.56 – 2.50 (m, 2H), 2.46 – 2.32 (m, 2H), 2.17 – 1.98 (m, 2H), 1.62 – 1.31 (m, 4H), 1.20 – 1.07 (m, 2H). ^{13}C NMR (151 MHz, $CDCl_3$) δ 184.61, 178.92, 170.85, 168.78, 168.76, 163.11, 159.75, 127.88, 100.08, 82.64, 75.47, 75.32, 71.02, 66.24, 65.72, 53.82, 36.00, 35.94, 35.58, 34.74, 28.76, 28.66. HRMS (ESI$^+$): m/z calculated for $C_{22}H_{30}F_2N_5O_{14}Pt^+$ ($[M+H]^+$): 821.1400, found: 821.1402.

Pt(IV)-Paclitaxel was obtained as white compound (45%). ^{1}H NMR (600 MHz, DMSO-d_6) δ 12.12 (s, 1H), 9.29 (d, 1H), 8.76 – 8.40 (m, 2H), 8.07 (d, *J* = 9.8 Hz, 2H), 7.97 (d, 2H), 7.79 (d, *J* = 7.6 Hz, 2H), 7.75 (t, *J* = 7.4 Hz, 1H), 7.68 (t, *J* = 7.6 Hz, 2H), 7.56 (t, *J* = 7.4 Hz, 1H), 7.49 (t, *J* = 7.5 Hz, 2H), 7.45 (t, *J* = 7.6 Hz, 2H), 7.38 (d, *J* = 7.6 Hz, 2H), 7.14 (t, *J* = 7.4 Hz, 1H), 6.30 (s, 1H), 5.81 (t, *J* = 9.2 Hz, 1H), 5.45 – 5.36 (m, 2H), 5.23 (d, *J* = 9.1 Hz, 1H), 4.91 (dd, *J* = 17.9, 8.3 Hz, 2H), 4.58 (s, 1H), 4.09 (m, 1H), 4.00 (q, *J* = 8.3 Hz, 2H), 3.54 (d, *J* = 7.1 Hz, 1H), 2.68 – 2.53 (m, 4H), 2.47 – 2.41 (m, 1H), 2.39 (m, 2H), 2.31 (m, 1H), 2.18 (s, 3H), 2.13 (m, 1H), 2.10 (s, 3H), 1.83 (s, 3H), 1.69 (m, 1H), 1.63 (t, *J* = 12.7 Hz, 1H), 1.52 (m, 2H), 1.49 (s, 3H), 1.43 (m, 1H), 1.41 – 1.35 (m, 1H), 1.29 (m, 1H), 1.21 – 1.14 (m, 2H), 1.02 (m, 6H). ^{13}C NMR (151 MHz, DMSO-d_6) δ 202.38, 178.76, 173.67, 170.13, 169.67, 168.71, 166.49, 165.24, 163.20, 163.08, 159.40, 139.54, 137.42, 134.37, 133.55, 133.38, 131.45, 129.91, 129.60, 128.76, 128.70, 128.40, 128.19, 127.54, 127.37, 83.56, 80.23, 76.72, 76.68, 75.26, 74.62, 74.45, 70.66, 70.50, 61.55, 60.33, 57.41, 54.34, 46.04, 45.73, 42.92, 36.50, 34.23, 31.00, 30.79, 30.17, 29.45, 26.39, 23.58, 23.30, 22.55, 21.40, 20.66, 14.24, 9.75, 8.63. HRMS (ESI$^+$): m/z calculated for $C_{60}H_{70}N_3O_{24}Pt^+$ ($[M+H]^+$): 1411.3992, found: 1411.3989.

Labeling Method

For all the radiolabeling of PSMA-617, 50 nmol PSMA-617 was used. For ^{68}Ga labeling, 9.5 mCi $^{68}GaCl_3$ in 0.6 M HCl (1 mL) was adjusted to pH 4.5 by using the solution of Na_2CO_3 (3 M) and NaOH (3 M); the mixture was incubated at 90°C for 10 min. For ^{86}Y labeling, 3.6 mCi $^{86}YCl_3$ in 0.1 M HCl (1 mL) was adjusted to pH = 4.5 by using the solution of Na_2CO_3 (3 M); the mixture was incubated at 90°C for 10 min. For ^{177}Lu labeling, 6.7 mCi $^{177}LuCl_3$ in 0.1 M HCl was added into the PSMA-617 in NaOAc buffer (0.2 M, pH = 4.5-5.0) and then incubated at 90°C for 15 min. pH-indicator strips were used to detect the pH of the labeling mixture. Sep-Pak Light C18 cartridges (Waters) activated by ethanol and water were used for purification, and the quality control was performed by using a radioactivity detector equipped with high-performance liquid chromatography (radio-HPLC).

The antibody PKU525 was labeled DFO or DOTA for further radiolabeling. For ^{89}Zr-labeling, 1 mg PKU525 was dilute by 250 μL Na_2CO_3-$NaHCO_3$ buffer (pH = 9). Then, 4.0 eq. of the chelator DFO-NCS dissolved in DMSO was added to the solution. After 1 h of incubation at 37°C, the DFO-PKU525 conjugates were purified with ultrafiltration centrifugal tubes. The purified antibody conjugate was added to the neutral ^{89}Zr solution, which was adjusted with 0.2 M sodium carbonate and 0.5 M HEPES buffer to pH 6.5-7.0 in advance. After incubating at 37°C for 1 h, the ^{89}Zr-PKU525 was purified through PD-10 chromatography (GE Health Care, USA).For ^{177}Lu labeling, 1 mg PKU525 was dilute by 250 μL Na_2CO_3-$NaHCO_3$ buffer (pH = 9) and 4.0 eq. of the chelator DOTA-NCS dissolved in DMSO was then added to the solution. After 1 h of incubation at 37°C, the DFO-PKU525 conjugates were purified with ultrafiltration centrifugal tubes. The ^{177}Lu solution was neutralized with 0.2 M sodium acetate buffer to pH 5.0 and the purified antibody conjugate was added to the solution. After incubating at 37°C for 1 h, the ^{177}Lu-PKU525 was purified through PD-10 chromatography. The labeling methods of ^{68}Ga and ^{86}Y

were consistent with that of ^{177}Lu.

Deoxygenation Method

Pre-treated solution, about 1 mL, was sealed in glass bottles with a rubber plug and a metal lid. All radionuclide-involved experiments were pretreated with freeze-thaw method to remove dissolved oxygen from the solution. Froze the solution and then pulled vacuum for few minutes while it was frozen until thawing the solution to room temperature.

Dose simulation

This simulation characterizes the irradiation geometry of a radioactive solution sample housed in a 15 mL Schlenk tube. The tube material is silicate, exhibiting a density of 2.5 g/cm^3. Within the tube, a hemispherical volume of water (1 mL) serves as the radioactive solution, with a diameter of 1.56 cm. The simulations were executed using Geant4, version 11.2.1, along with the FTFP_BERT_HP Physical Process Package.

Biological Methods

Cell culture

HT1080-FAP was grown in MEM (Minimum Essential Medium). 4T1 and 4T1-FAP were grown in RPMI-1640 (Roswell Park Memorial Institute-1640). HEK293T was grown in DMEM (Dulbecco's modified Eagle medium). Culture medium contained 10%(v/v) FBS (fetal bovine serum), 1% penicillin (100 U/mL)/streptomycin (100 µg/mL). All cell cultures were incubated at 37°C under 5% CO_2 and the medium was changed each 2-3 days. When the cell confluency reached 80%, trypsin-EDTA solution was used for cell subculturing and other experiments.

Cell viability assays

Cell viability was assessed with the CCK-8 assay following the protocol. Each assay was repeated three times. To detect the cytotoxicity of Gemcitabine and Pt(IV)-Gem, 4T1-FAP cells were seeded in a 96-well plate at a concentration of 1×10^4 /mL in 100 µL of RMPI-1640 medium with 10% FBS and 1% penicillin/streptomycin, and maintained at 37°C in a 5% CO_2 incubator for 24 h. Afterwards, the medium of each well was replaced by fresh medium containing a final concentration of 0.5 mg/mL CCK-8. The plates were incubated at 37°C under 5% CO_2 for 2 h. The absorbance was measured at 450 nm. The absorbance of treated cells was compared with the absorbance of the control group, of which the viability was set as 100%.

Cellular uptake of ^{177}Lu-PKU525

The cells were typically seeded at a density of 2×10^5 cells/mL in 24-well plates and incubated in an incubator containing 5% CO_2 at 37°C for 12 h. 250 µL RMPI-1640 medium and different dose of ^{177}Lu-PKU525 solution was added to the adherent cells. After 2 h, the medium was removed and the cells were washed twice with PBS. The cells were then lysed with NaOH (1 mol/L, 0.5 mL) and washed twice with PBS (0.5 mL). The radioactive counts were detected from the collected NaOH (0.5 mL) and PBS (0.5 mL × 2) solution with WIZARD2 2480 Automatic Gamma Counter (PerkinElmer Instruments Inc.).

Confocal imaging

The cells were typically seeded at a density of 5×10^4 cells/mL in an 8-well confocal dish and incubated in an incubator containing 5% CO_2 at 37°C for 12 h. 100 µL RMPI-1640 medium and different dose of ^{177}Lu-PKU525 solution was added to the adherent cells. After 2 h, the medium was removed, followed

by PBS wash and addition of Pt(IV)-HD solution (5 μM final concentration). Then the cells were incubated with AnaeroPack (Mitsubishi Gas Chemical Company Inc.) at 37°C to build hypoxic environment before drawing out the medium and adding 100 μL RMPI-1640 medium. The fluorescent images were taken by a laser scanning confocal microscope (Nikon, Japan) and the excitation wavelength was 638 nm. For the imaging quantification, all cells on the images were set as region of interest, and the fluorescence intensity was measured by Nikon image software (version 4.0).

Immunofluorescence assay for cellar DNA injury

4T1-FAP cells were seeded in an 8-well confocal dish at a concentration of 5×10^4 cells/mL and maintained at 37°C in a 5% CO_2 incubator for 12 h. After incubation with ^{177}Lu-PKU525 and addition of the medium containing 5 μM Pt(IV)-Gem in hypoxic environment for 24 h, the solution was removed and the cells were washed three times with PBS. The cells were fixed with paraformaldehyde for 30 min and were washed again with PBS. After blocked with 0.5% Triton-100 and 2% goat serum in PBS for 1 hour, the cells were incubated with anti-γH2AX (phosphorylation site S139) antibody at 4°C overnight. Then the cells were incubated with secondary antibody Goat anti-rabbit IgG H&L (Alexa Fluor® 647) at a concentration of 1 μg/mL for 1 hour. After washing the cells with PBST for three times, DAPI staining was added. The resulting cells were observed under a confocal microscope with excitation wavelengths of 405 nm and 638 nm.

Measurement of oxaliplatin concentrations in cells

4T1-FAP cells were seeded in a 6-well plate and grew to 70% confluence. The cells were incubated with ^{177}Lu-PKU525 and then added the medium containing 5 μM Pt(IV)-Gem in hypoxic environment for 12 h. Removed the supernatant and added the fresh medium. The cells were incubated for 16 h and then treated with trypsin. The cells were collected and washed twice with cold PBS. Genomic DNA was extracted using a mammalian genomic DNA extraction kit. The amount of DNA was quantified using Nanodrop. Then the genomic DNA was digested with 65% nitric acid overnight. The solution was diluted to a final volume of 1 mL and the concentration of platinum was determined by ICP-MS. The level of platinum on DNA was shown in pg Pt/μg DNA.

Animal model

All animal care and experimental procedure were performed by following the animal protocols (CCME-LiuZB-2) approved by the ethics committee of Peking University. 6~8 weeks old female BALB/c mice were ordered from Vital River Laboratories (Beijing, China) and kept under Specific Pathogen Free (SPF) condition with free access to standard food and water, with the bedding replaced once every 3 days. After 1 week, approximately 1×10^6 4T1-FAP cells suspended in 100 μL of PBS were implanted subcutaneously into the right shoulder of BALB/c mice to build the tumor-bearing mice. To build the metastasis model, 5×10^5 4T1-FAP cells suspended in 100 μL of PBS were injected to BALB/c mice by intravenous administration.

Fluorescence image of mice

^{177}Lu-PKU525 with different radio activity was injected into mice by intravenous administration. After 48 h, the DSPE-PEG encapsulated Pt(IV)-HD (20 μL, 10 μM) was injected into mice by intravenous injection. The fluorescence images of mice were acquired on the IVIS lumina II imaging system using a 640 nm excitation light at a fluorescence emission window of about 710 nm after 24 h. Tumor and the major organs including the heart, liver, spleen, lung and kidney were excised from mice and the fluorescence images were taken.

PET image of mice

Approximately, 200 μCi ^{89}Zr-antibody was injected into mice by intravenous administration. At the appropriate time point, the 15-minute whole-body PET imaging was collected under isoflurane anesthesia. The collected data were then dynamically reconstructed through the data post-processing workstation. Tera-Tomo 3D method and Variance Reduced D.W. were used during the reconstruction, performing random correction and scattering elimination. For each acquisition, the region of interest (ROI) was acquired on the decay-corrected reconstructed image by Nucline NanoScan software (InterView^{TM} FUSION, Mediso Medical Imaging Systems). Radioactivity concentration in tissue was obtained from the average and maximum SUV value of the ROI.

SPECT image of mice

Approximately, 200 μCi ^{177}Lu-antibody was injected into mice by intravenous administration. At the appropriate time point, the 15-minute whole-body SPECT imaging was collected under isoflurane anesthesia. The collected data were then dynamically reconstructed through the data post-processing workstation. Standard data acquisition and image reconstruction of the SPECT data were performed, generating maximum intensity projection images. Volumes of the tumor and major organs were delineated, from which the tracer uptake was calculated in the unit of %ID g^{-1}. The data analysis was performed on Interview Fusion software (v.3.09.008.0000).

Biodistribution of Pt

Tumor-bearing mice were injected with Pt(IV)-Gem (20 mg/kg) by intravenous administration. The mice were then sacrificed at preset time points post injection. Blood, livers, kidneys and tumors were collected and weighted before digested with HNO_3 using a microwave accelerated reaction system. Afterwards, the digestion solution was diluted with deionized water. The final Pt concentration was determined by ICP-MS.

Treatment of mice

When the tumor volume reached approximately 50 mm^3, tumor-bearing mice were randomly divided into 4 groups including treatment groups and treated by PBS, Pt(IV)-Gem, ^{177}Lu-PKU525 antibody, ^{177}Lu-PKU525 + Pt(IV)-Gem. In the groups receiving radiopharmaceutical administration, 0.1 mCi ^{177}Lu-PKU525 was injected per mouse on Day 0. On Day 2 and 4, 20 mg/kg Pt(IV)-Gem was given to initiate the combination therapy.

The mice were weighted every 2 days and the tumor long diameter (L) and short diameter (W) were measured with a caliper at the same time; the tumor volume was determined using the volume formula for an ellipsoid (that is, $1/2 \times L \times W^2$). When the tumor size reached 1500 mm^3 or the loss was > 20% of total body weight, the mice were removed from the experimental group and euthanized. The tumors were dissected from the surrounding fascia, weighted, minced into pieces by sterile scissors.

H&E staining

The major organs including the heart, liver, spleen, lung and kidney were excised from mice of different groups 21 days after treatment for the histopathologic study. Organs were fixed in 4% paraformaldehyde, embedded with paraffin, sectioned into slices, and stained with hematoxylin and eosin. Samples were chosen at random, and the slices were photographed by 3Dhistech.

Reference

(1) Wang, K.; Xiao, X.; Jiang, M.; Li, J.; Zhou, J.; Yuan, Y. An NIR-Fluorophore-Based Theranostic for

Selective Initiation of Tumor Pyroptosis-Induced Immunotherapy. *Small* **2021**, *17* (36), 2102610.
(2) Xu, Z.; Chan, H. M.; Li, C.; Wang, Z.; Tse, M.-K.; Tong, Z.; Zhu, G. Synthesis, structure, and cytotoxicity of oxaliplatin-based platinum (IV) anticancer prodrugs bearing one axial fluoride. *Inorg. Chem.* **2018**, *57* (14), 8227-8235.

Supplementary Tables

Table S1. Basic nuclear physical properties of tested radionuclides. Data sources refer to International Atomic Energy Agency (IAEA) Nuclear Data Services website https://www-nds.iaea.org.

Radionuclide	Mean energy of major radiation (keV)	Intensity %	Half Life (h)	Decay constant (s^{-1})
^{18}F	249.8 (β+)<br>511 (γ from annihilation)	96.73<br>≈ 193.4	1.828	$1.053*10^{-4}$
^{68}Ga	836.0 (β+)<br>352.6 (β+)<br>1077.3 (γ)<br>511 (γ from annihilation)	87.72<br>1.19<br>3.22<br>≈ 178	1.128	$1.707*10^{-4}$
^{86}Y	535.4 (β+)<br>681.1 (β+)<br>883.3 (β+)<br>1436.8 (β+)<br>452.6 (β+)<br>767.8 (β+)<br>509.4 (β+)<br>394.1 (β+)<br>1077.6 (β+)<br>1076.63 (γ)<br>627.7 (γ)<br>1153.0 (γ)<br>777.4 (γ)<br>1920.8 (γ)<br>1854.4 (γ)<br>443.13 (γ)<br>703.3 (γ)<br>511 (γ from annihilation)	11.9<br>5.6<br>3.6<br>2.0<br>1.9<br>1.7<br>1.33<br>1.1<br>0.9<br>93.8<br>32.6<br>30.5<br>22.4<br>20.8<br>17.16<br>16.9<br>15.4<br>≈ 64	14.74	$1.306*10^{-5}$
^{89}Zr	396 (β+)<br>909.15 (γ)<br>511 (γ from annihilation)	22.74<br>99.04<br>≈ 45	78.36	$2.457*10^{-6}$
^{177}Lu	148.8 (β-)<br>112.2 (β-)<br>47.23 (β-)<br>208.4 (γ)<br>112.9 (γ)	79.44<br>8.89<br>11.66<br>10.41<br>6.23	159.4	$1.208*10^{-6}$

Table S2. Simulated energy deposit of tested radionuclides. Initial energy deposit of corresponding

Radionuclide	Energy deposit per particle (keV)	Energy deposit per decay (keV)	Dose per hour with no decay (Gy/h)	Energy deposit at the indicated time (Gy)								
				1 h	2 h	6 h	12 h	24 h	48 h	96 h	168 h	∞
^{18}F	89.82	260.6	5.561	4.628	7.796	13.16	14.51	14.66	14.67	14.67	14.67	14.67
^{68}Ga	216.9	585.8	12.50	9.340	14.39	19.83	20.33	20.34	20.34	20.34	20.34	20.34
^{86}Y	57.47	197.5	4.214	4.116	8.044	22.03	38.64	60.62	80.23	88.63	89.58	89.61
^{89}Zr	59.47	99.18	2.117	2.107	4.196	12.37	24.10	45.77	82.78	136.9	185.1	239.3
^{177}Lu	112.6	131.3	2.802	2.796	5.580	16.59	32.76	63.86	121.4	219.9	334.0	644.4

An example for the calculation of the energy deposit of Lu-177 in 1 h:

Energy deposit per particle (calculate by Geant4):

$$E_0 = 112.6 \text{ keV}$$

The average particles number per decay

$$n = 79.44\% + 8.89\% + 11.66\% + 10.41\% + 6.23\% = 1.166$$

So, the total energy deposit per decay is

$$E = E_0 \times n = 112.6 \text{ keV} \times 1.166 = 131.3 \text{ keV} = 2.104\times10^{-14} \text{ J}$$

If no decay decrease is considered, total energy deposit rate for 1 mCi (37 MBq) Lu-177 in 1 mL H_2O after 1 h incubation is

$$\dot{D} = 2.1037\times10^{-14} \text{ J} \times 3.7\times10^{7} \text{ Bq} \times 3600 \text{ s} / 0.001 \text{ kg} = 2.802 \text{ Gy/h}$$

Considering decay decrease, the energy deposit in t hours is

$$\int_0^t \dot{D}\times\left(\frac{1}{2}\right)^{\frac{t'}{t_{1/2}}} dt' = \frac{\dot{D} \times t_{1/2} \times (1-2^{\frac{-t}{t_{1/2}}})}{\ln 2}$$

Thus, the energy deposit of Lu-177 in 1 hour is

$$D = \frac{2.802 \text{ Gy/h} \times 159.4 \text{ h} \times (1 - 2^{\frac{-1 \text{ h}}{159.4 \text{ h}}})}{\ln 2} = 2.796 \text{ Gy}$$

Supplementary Figures

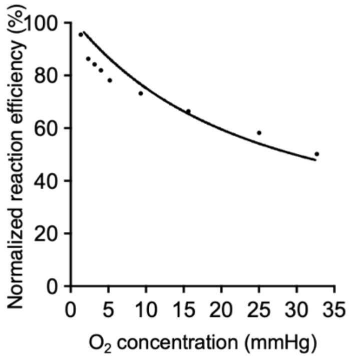

Figure S1. The correlation between radionuclide-induced reduction yield and oxygen concentration under the incubation with 3 mCi/mL [^{68}Ga]Ga-PSMA-617 for 12h.

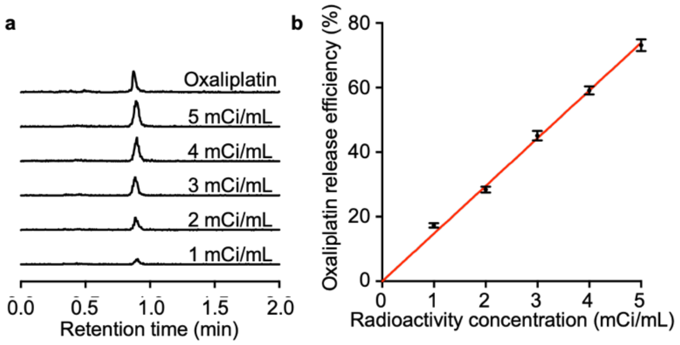

Figure S2. The (a) UPLC-MS ([M+H$^+$] = 397) and (b) quantification of release oxaliplatin from Pt(IV)-Cou under different radioactivity concentration of [^{68}Ga]Ga-PSMA-617 in PBS for 12 h at room temperature (n = 3 independent experiments).

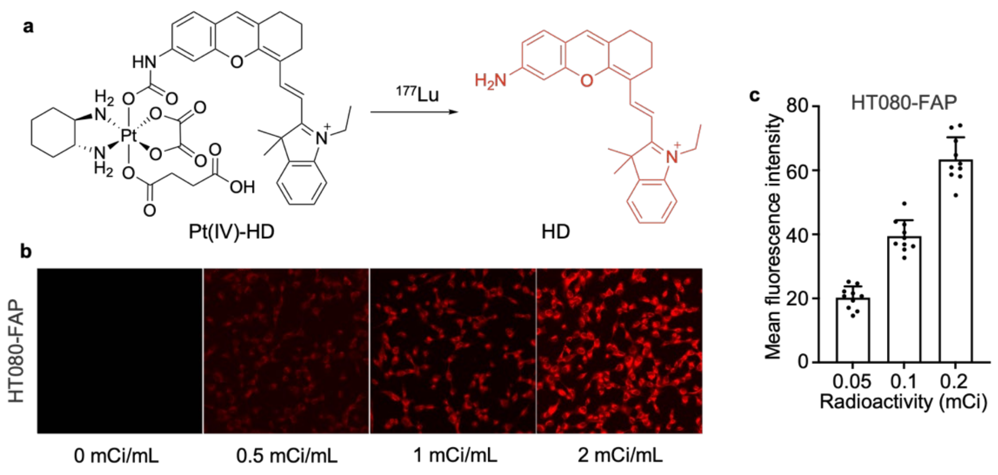

Figure S3. (a) Schematic representation of radionuclide-induced release of hemicyanine dye (HD) from Pt(IV)-HD as model reaction. (b) Confocal fluorescence images of HT1080-FAP cells after being treated with a radioactivity gradient of [^{177}Lu]Lu-PKU525 (λ_{ex} = 638 nm) and (c) fluorescence intensity analysis for each group (λ_{ex}= 638 nm), scale bar = 25 μm, *n* = 10 fields of view.

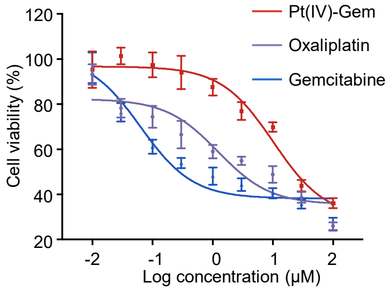

Figure S4. Cell viability assays of 4T1-FAP cells incubated with oxaliplatin, gemcitabine and Pt(IV)-Gem at variant concentration, respectively, for 24 h, n = 5 samples.

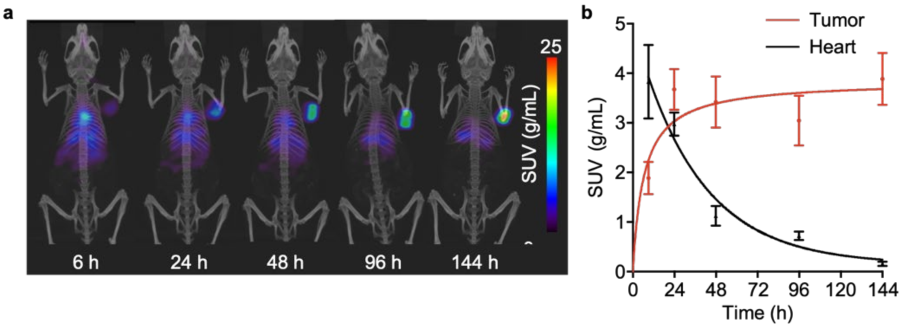

Figure S5. (a) Dynamic SPECT-CT images of 4T1-FAP tumor-bearing mice at the indicated time after intravenous administration of [^{177}Lu]Lu-PKU525. (b) Time-activity curve of [^{177}Lu]Lu-PKU525 in blood and tumor, n = 3 mice.

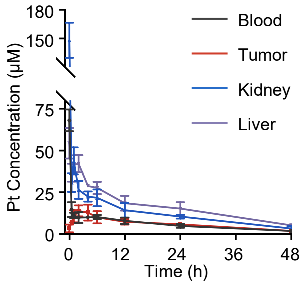

Figure S6. Biodistribution of Pt(IV)-Gem in different major organs in the time span of 48 h intravenous injection, n = 3 mice

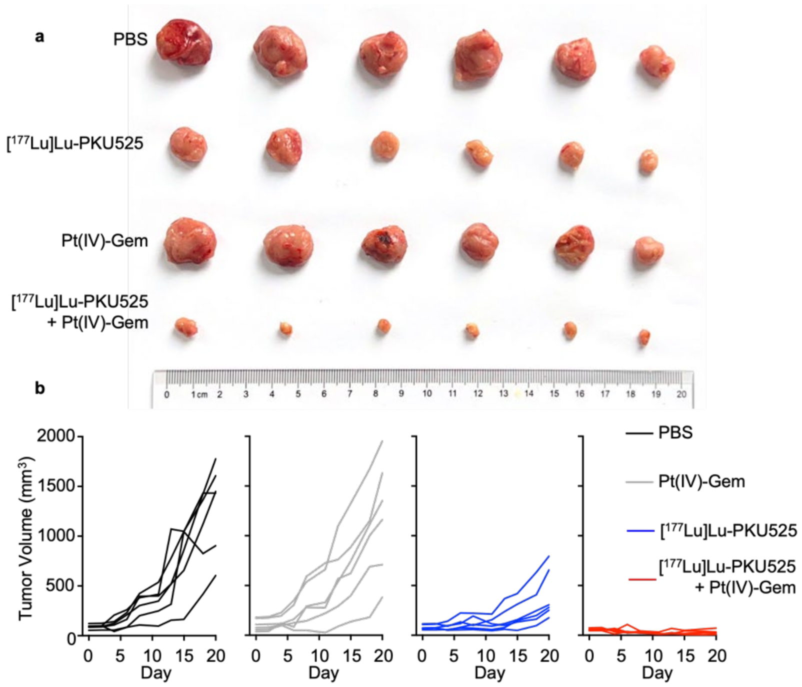

Figure S7. (a) The photograph of tumor after the indicated treatments (n = 6 mice). (b) Individual growth of 4T1-FAP tumors after the indicated treatments (n = 6 mice).

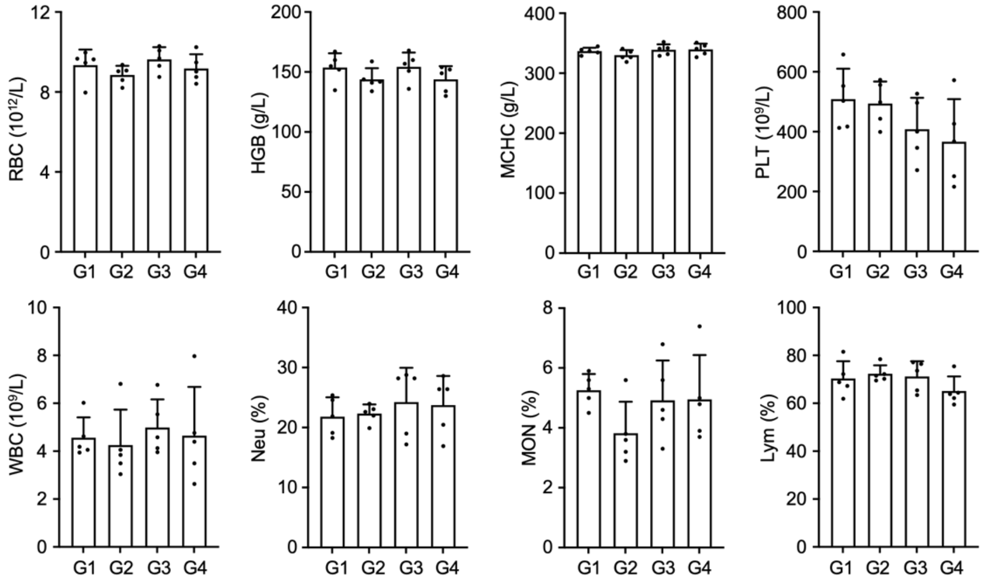

Figure S8. Complete blood panel analysis to evaluate the toxicology of treatment strategies in blood after the indicated treatment (n = 5 mice). G1: PBS; G2: [^{177}Lu]Lu-PKU525; G3: Pt(IV)-Gem; G4: [^{177}Lu]Lu-PKU525 + Pt(IV)-Gem.

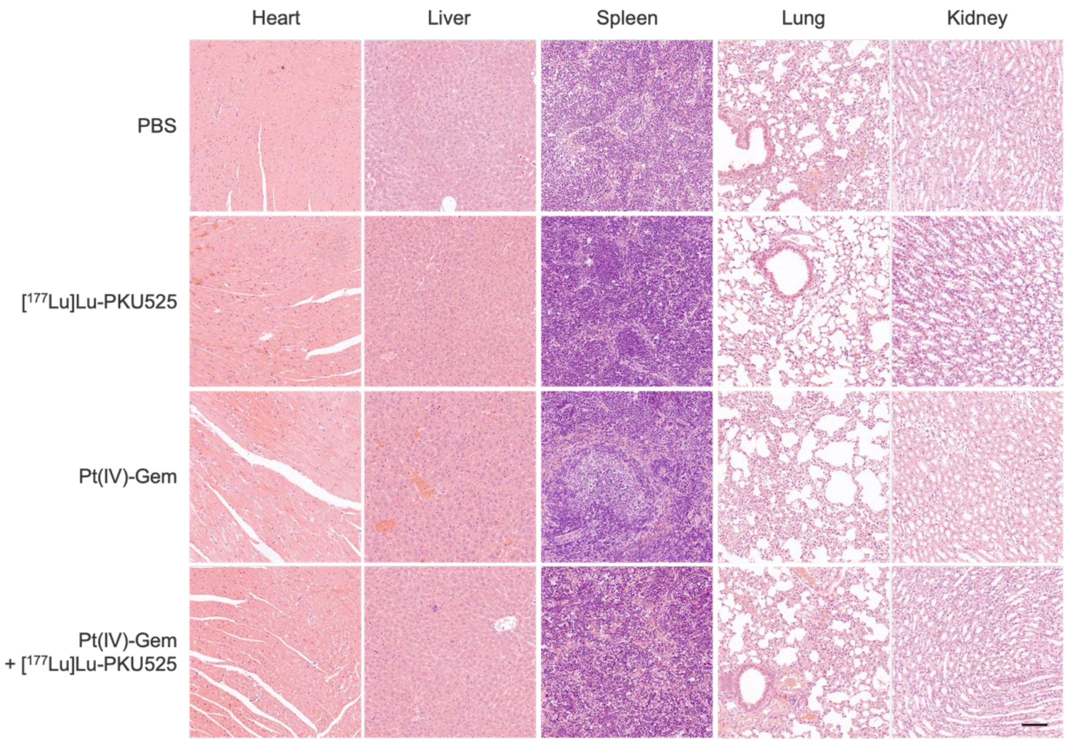

Figure S9. Representative H&E staining of major organs to evaluate the toxicology of treatment strategies in blood after the indicated treatment, scale bar = 100 μm.

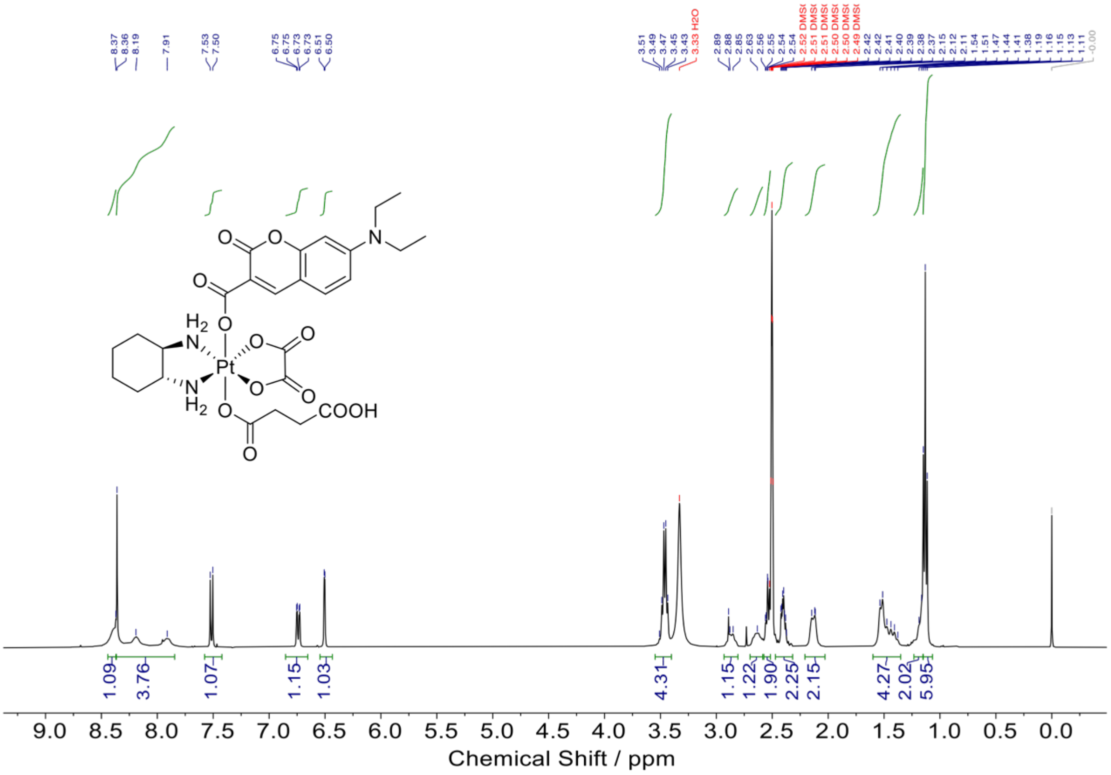

Figure S10. ^{1}H NMR spectrum of Pt(IV)-Cou.

Figure S11. ^{13}C NMR spectrum of Pt(IV)-Cou.

Peking University Mass Spectrometry Sample Analysis Report

Analysis Info

Analysis Name	FTMS-23030124_Pos_20230310_000001.d	Acquisition Date	3/10/2023 2:51:40 PM
Sample	Pt-coumarin	Instrument	Bruker Solarix XR FTMS
Comment		Operator	Peking University

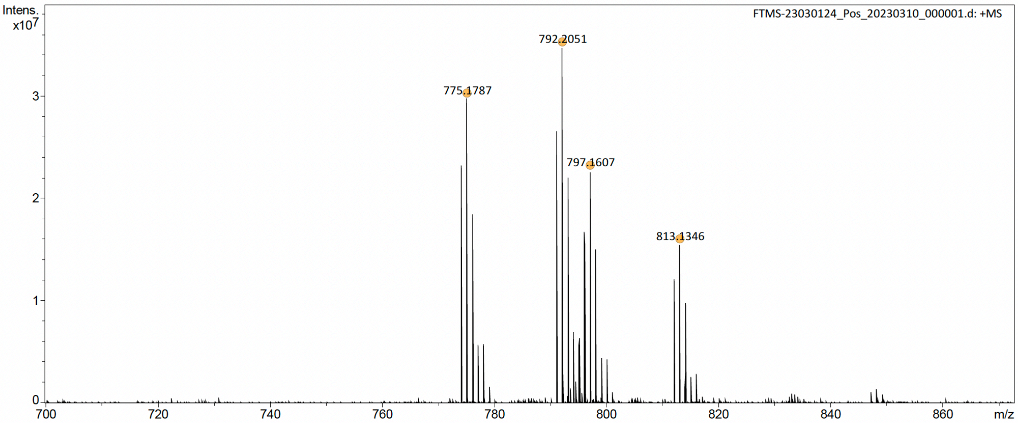

Meas. m/z	#	Ion Formula	Score	m/z	err [ppm]	Mean err [ppm]	mSigma	rdb	e⁻ Conf	N-Rule
775.1787	1	C26H34N3O12Pt	100.00	775.1785	0.1	-0.3	86.4	14.0	even	-
792.2051	1	C26H37N4O12Pt	100.00	792.2050	0.2	0.4	77.0	13.0	even	-
797.1607	1	C26H33N3NaO12Pt	100.00	797.1604	-0.0	0.0	78.0	14.0	even	-
813.1346	1	C26H33KN3O12Pt	100.00	813.1344	0.0	-0.9	118.7	14.0	even	-

Figure S12. High-resolution mass spectrum of Pt(IV)-Cou.

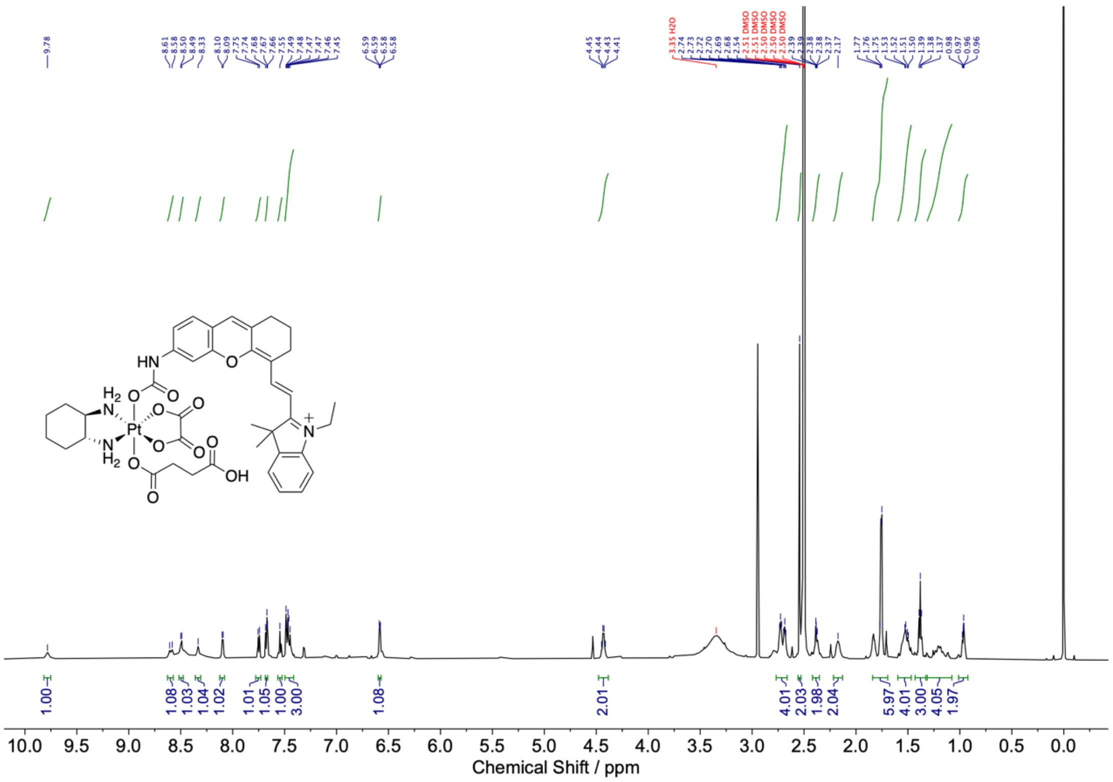

Figure S13. ^{1}H NMR spectrum of Pt(IV)-HD.

Peking University Mass Spectrometry Sample Analysis Report

Analysis Info

Analysis Name	FTMS-24110017_Pos_20241104_000006.d	Acquisition Date	11/4/2024 1:49:02 PM
Sample	PtHD	Instrument	Bruker Solarix XR FTMS
Comment		Operator	Peking University

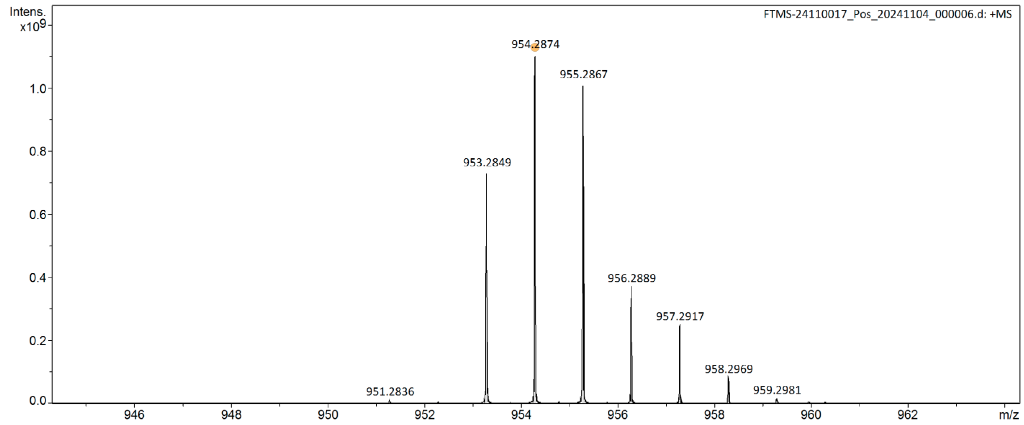

Meas. m/z	#	Ion Formula	Score	m/z	err [ppm]	Mean err [ppm]	mSigma	rdb	e⁻ Conf	N-Rule
954.2874	1	C40H47N4O11Pt	100.00	954.2884	1.0	4.5	6.2	22.0	even	-

Figure S14. High-resolution mass spectrum of Pt(IV)-HD.

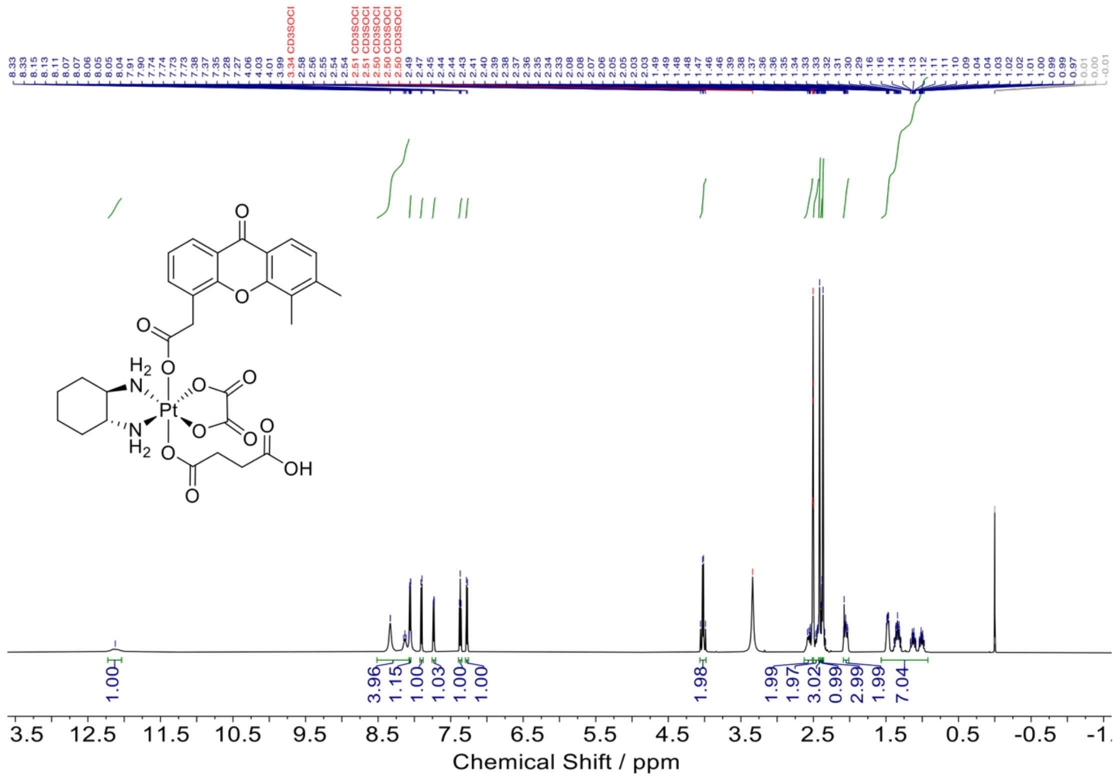

Figure S15. ^{1}H NMR spectrum of Pt(IV)-Vadimezan.

Figure S16. ^{13}C NMR spectrum of Pt(IV)-Vadimezan.

Peking University Mass Spectrometry Sample Analysis Report

Analysis Info

Analysis Name	FTMS-23050117_Pos_20230520_000010.d	Acquisition Date	5/20/2023 2:52:16 PM
Sample	Pt-Vadimezan	Instrument	Bruker Solarix XR FTMS
Comment		Operator	Peking University

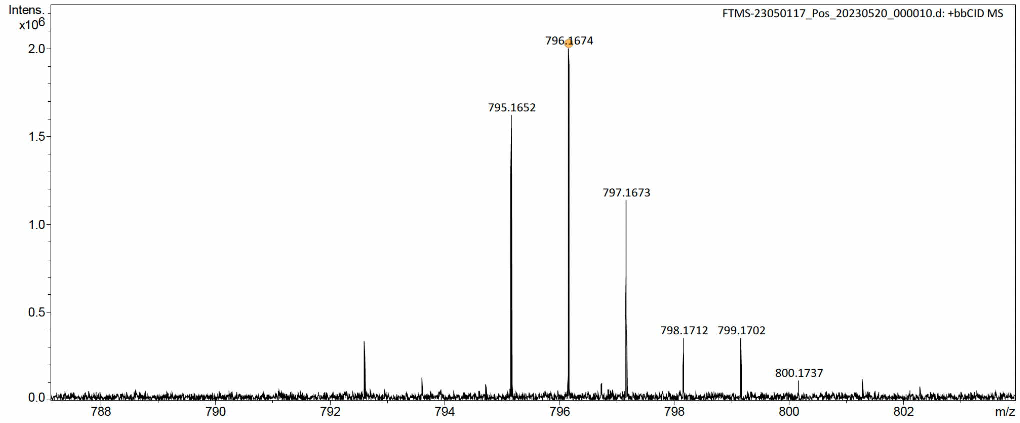

Meas. m/z	#	Ion Formula	Score	m/z	err [ppm]	Mean err [ppm]	mSigma	rdb	e⁻ Conf	N-Rule
796.1674	1	C29H33N2O12Pt	100.00	796.1676	0.6	0.8	114.5	17.0	even	-

Figure S17. High-resolution mass spectrum of Pt(IV)-Vadimezan.

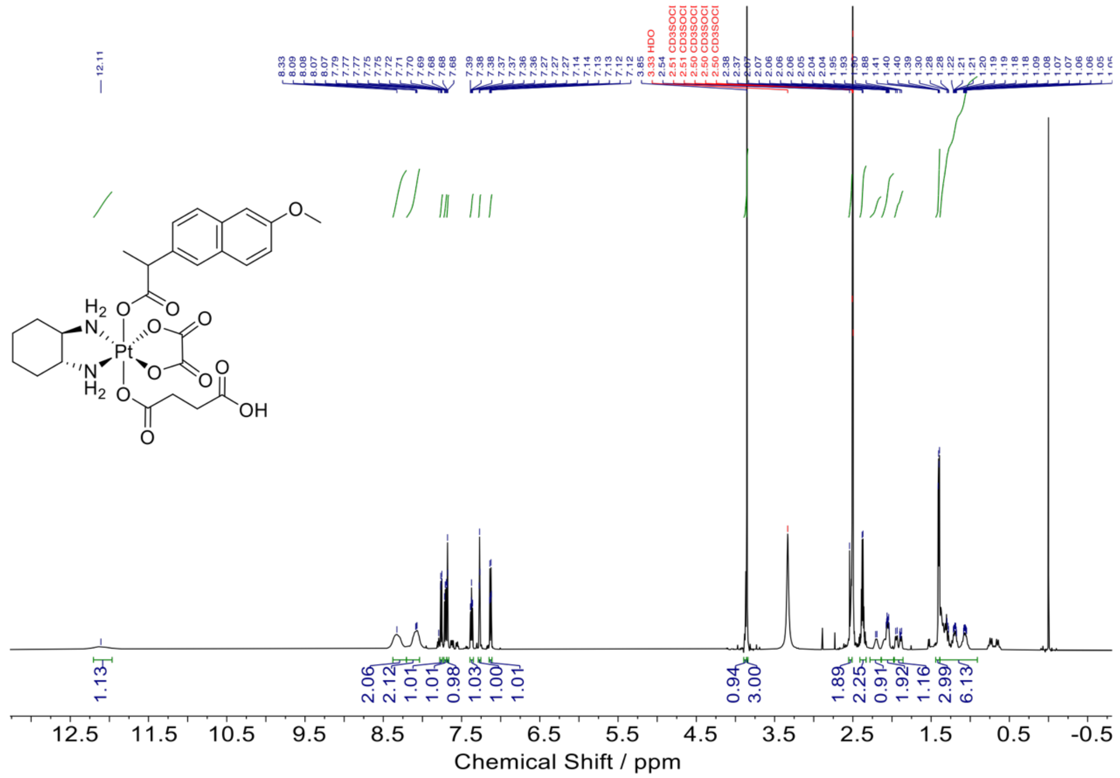

Figure S18. ^{1}H NMR spectrum of Pt(IV)-Naproxen.

Figure S19. ^{13}C NMR spectrum of Pt(IV)-Naproxen.

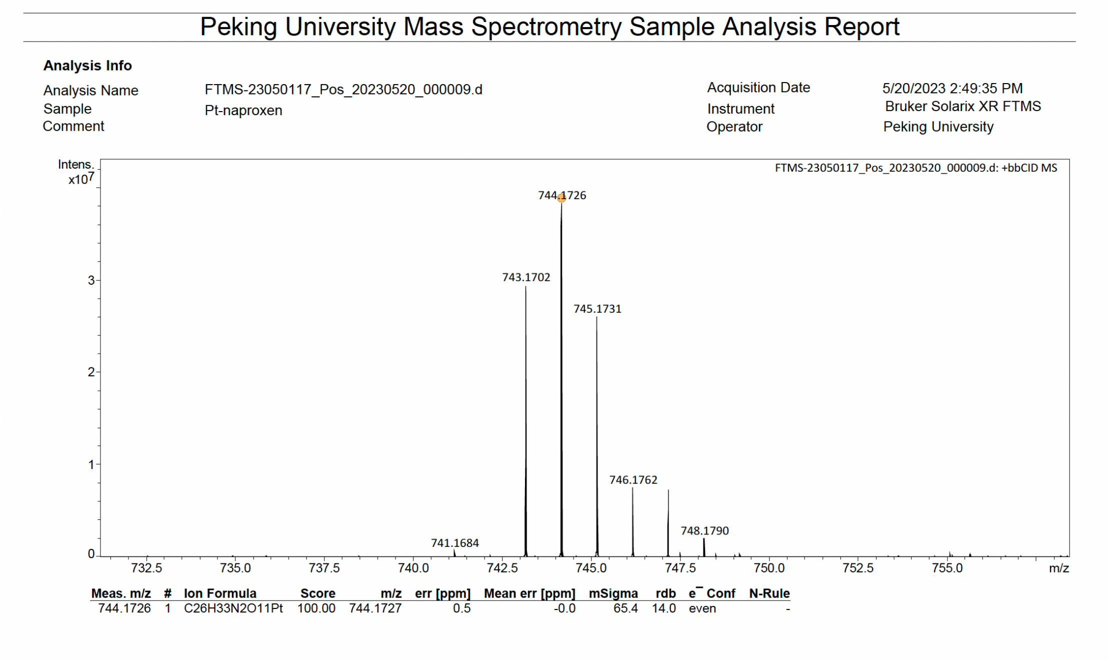

Figure S20. High-resolution mass spectrum of Pt(IV)-Naproxen.

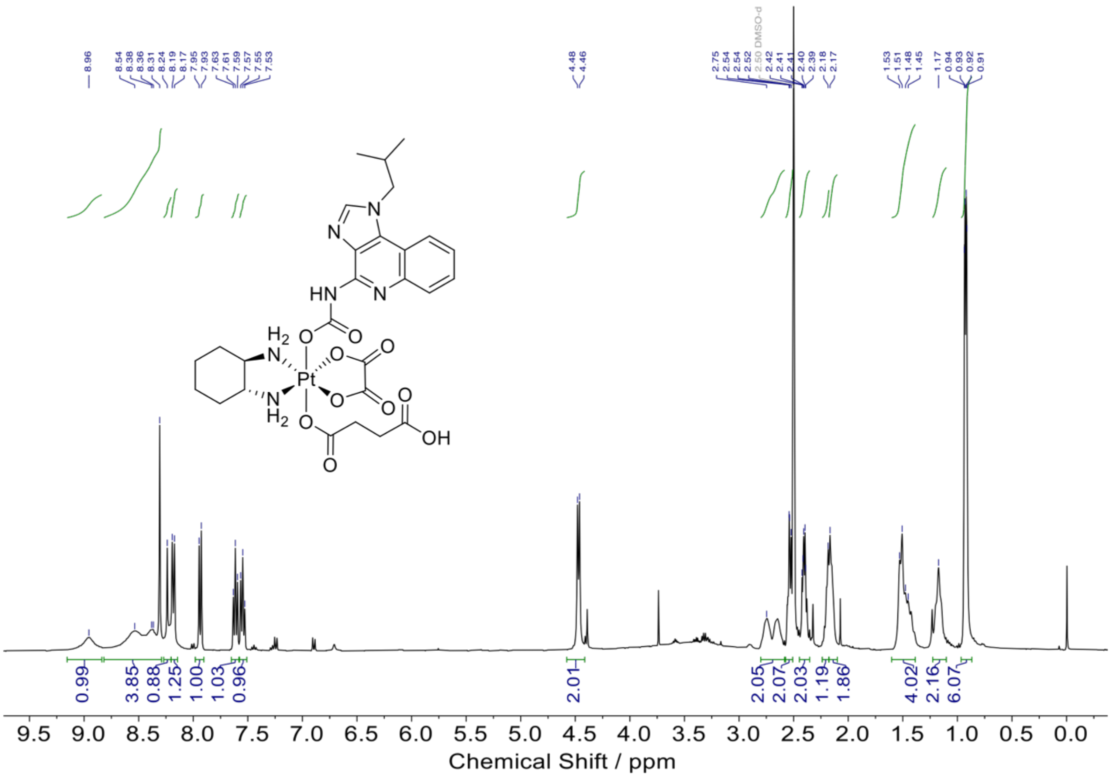

Figure S21. ^1H NMR spectrum of Pt(IV)-Imiquimod.

Figure S22. ^{13}C NMR spectrum of Pt(IV)-Imiquimod.

Peking University Mass Spectrometry Sample Analysis Report

Analysis Info

Analysis Name	FTMS-23030124_Pos_20230310_000013.d	Acquisition Date	3/10/2023 3:13:20 PM
Sample	Pt-imiquimod	Instrument	Bruker Solarix XR FTMS
Comment		Operator	Peking University

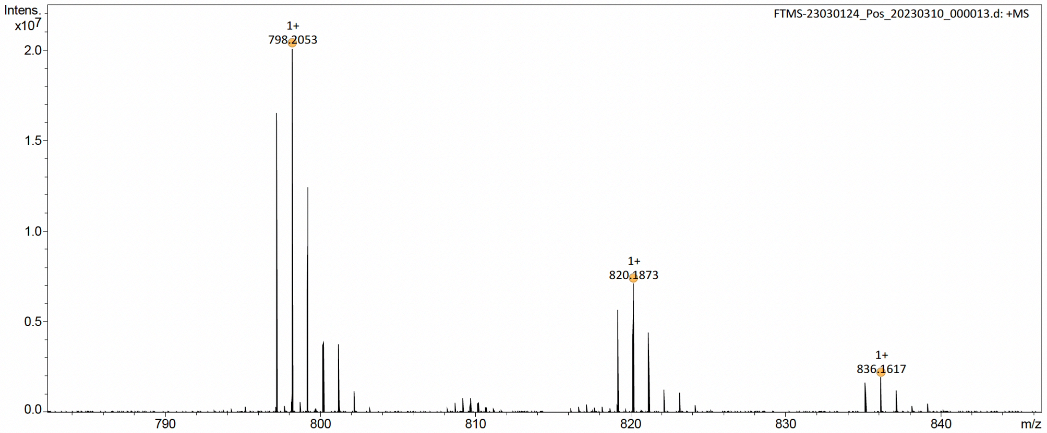

Meas. m/z	#	Ion Formula	Score	m/z	err [ppm]	Mean err [ppm]	mSigma	rdb	e⁻ Conf	N-Rule
798.2053	1	C27H35N6O10Pt	100.00	798.2057	0.7	1.2	88.8	16.0	even	-
820.1873	1	C27H34N6NaO10Pt	100.00	820.1877	0.7	0.6	95.8	16.0	even	-
836.1617	1	C27H34KN6O10Pt	100.00	836.1616	0.2	0.0	120.0	16.0	even	-

Figure S23. High-resolution mass spectrum of Pt(IV)-Imiquimod.

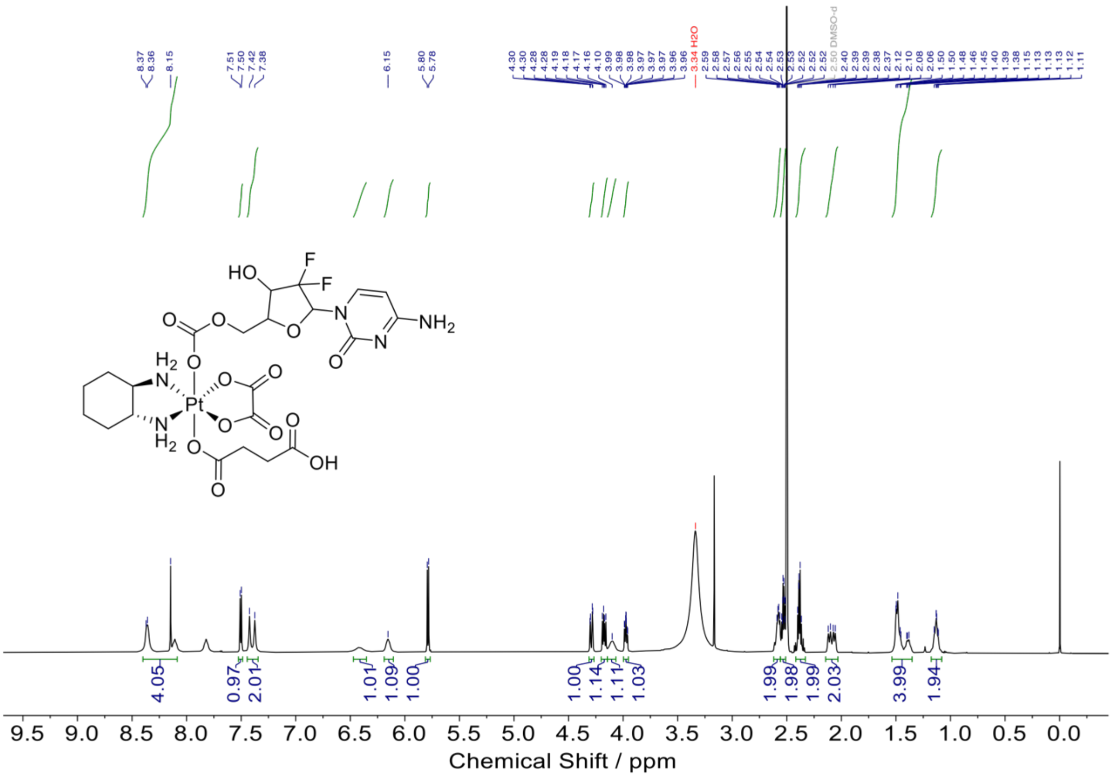

Figure S24. ^{1}H NMR spectrum of Pt(IV)-Gem.

Figure S25. ^{13}C NMR spectrum of Pt(IV)-Gem.

Peking University Mass Spectrometry Sample Analysis Report

Analysis Info

Analysis Name	FTMS-23050117_Pos_20230520_000008.d	Acquisition Date	5/20/2023 2:46:23 PM
Sample	Pt-gemcitabine	Instrument	Bruker Solarix XR FTMS
Comment		Operator	Peking University

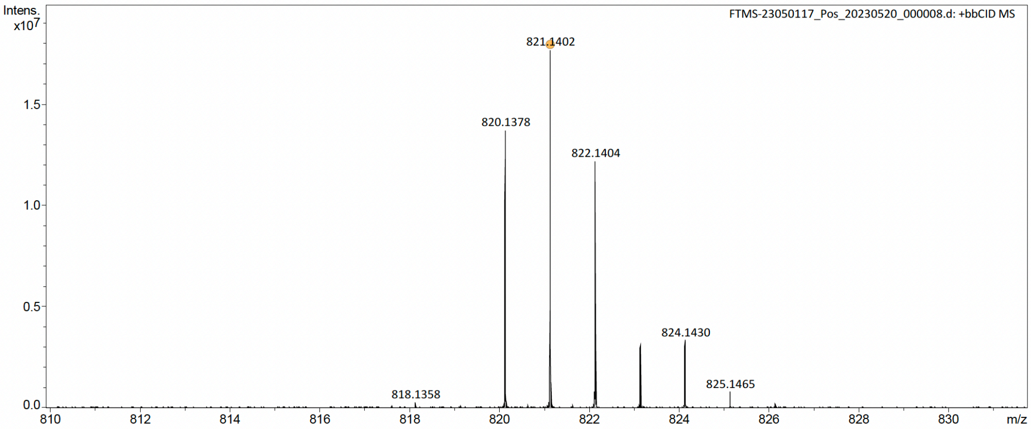

Meas. m/z	#	Ion Formula	Score	m/z	err [ppm]	Mean err [ppm]	mSigma	rdb	e⁻ Conf	N-Rule
821.1402	1	C22H30F2N5O14Pt	100.00	821.1400	-0.1	0.2	59.9	12.0	even	-

Figure S26. High-resolution mass spectrum of Pt(IV)-Gem.

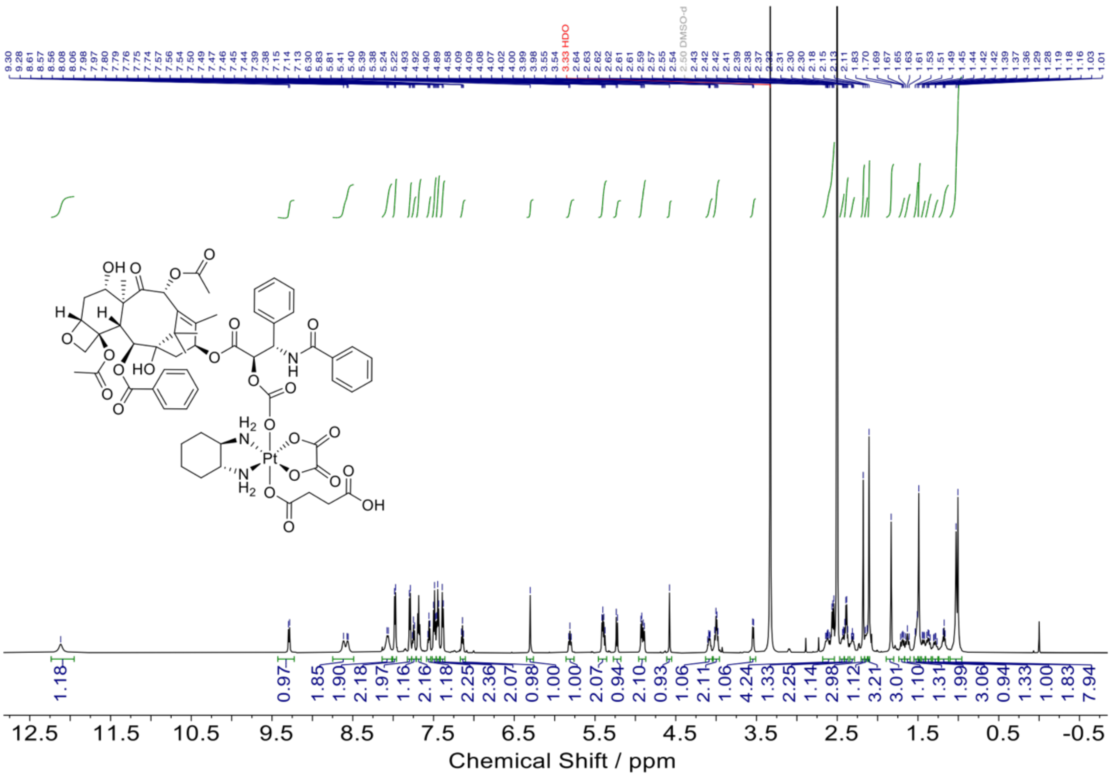

Figure S27. ^{1}H NMR spectrum of Pt(IV)-Paclitaxel.

Figure S28. ^{13}C NMR spectrum of Pt(IV)-Paclitaxel.

Peking University Mass Spectrometry Sample Analysis Report

Analysis Info

Analysis Name	FTMS-24030021_Pos_20240306_000006.d	Acquisition Date	3/6/2024 5:37:45 PM
Sample	Pt-Paclitaxel	Instrument	Bruker Solarix XR FTMS
Comment		Operator	Peking University

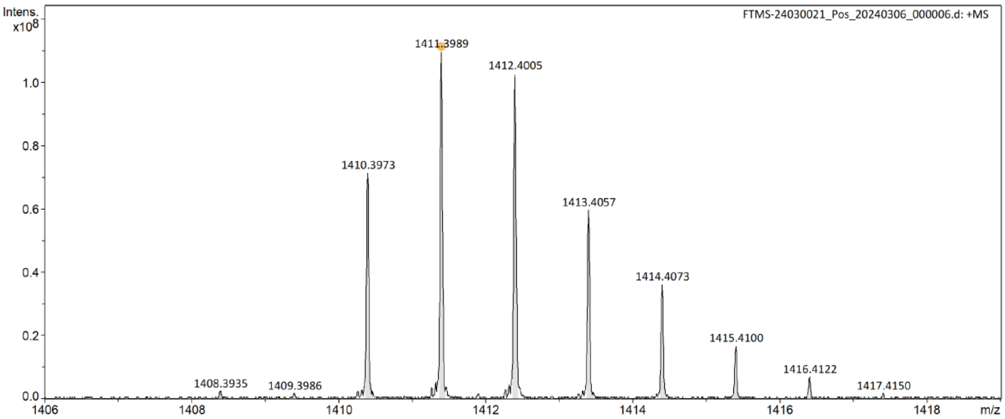

Meas. m/z	#	Ion Formula	Score	m/z	err [ppm]	Mean err [ppm]	mSigma	rdb	e⁻ Conf	N-Rule
1411.3989	1	C60H70N3O24Pt	100.00	1411.3992	0.5	-0.5	36.3	30.0	even	-

Figure S29. High-resolution mass spectrum of Pt(IV)-Paclitaxel.
